# Spatio-Temporal Migration Patterns of Pacific Salmon Smolts in Rivers and Coastal Marine Waters

**DOI:** 10.1371/journal.pone.0012916

**Published:** 2010-09-23

**Authors:** Michael C. Melnychuk, David W. Welch, Carl J. Walters

**Affiliations:** 1 Department of Zoology and Fisheries Centre, University of British Columbia, Vancouver, British Columbia, Canada; 2 Kintama Research Corporation, Nanaimo, British Columbia, Canada; University of Canterbury, New Zealand

## Abstract

**Background:**

Migrations allow animals to find food resources, rearing habitats, or mates, but often impose considerable predation risk. Several behavioural strategies may reduce this risk, including faster travel speed and taking routes with shorter total distance. Descriptions of the natural range of variation in migration strategies among individuals and populations is necessary before the ecological consequences of such variation can be established.

**Methodology/Principal Findings:**

Movements of tagged juvenile coho, steelhead, sockeye, and Chinook salmon were quantified using a large-scale acoustic tracking array in southern British Columbia, Canada. Smolts from 13 watersheds (49 watershed/species/year combinations) were tagged between 2004–2008 and combined into a mixed-effects model analysis of travel speed. During the downstream migration, steelhead were slower on average than other species, possibly related to freshwater residualization. During the migration through the Strait of Georgia, coho were slower than steelhead and sockeye, likely related to some degree of inshore summer residency. Hatchery-reared smolts were slower than wild smolts during the downstream migration, but after ocean entry, average speeds were similar. In small rivers, downstream travel speed increased with body length, but in the larger Fraser River and during the coastal migration, average speed was independent of body length. Smolts leaving rivers located towards the northern end of the Strait of Georgia ecosystem migrated strictly northwards after ocean entry, but those from rivers towards the southern end displayed split-route migration patterns within populations, with some moving southward.

**Conclusions/Significance:**

Our results reveal a tremendous diversity of behavioural migration strategies used by juvenile salmon, across species, rearing histories, and habitats, as well as within individual populations. During the downstream migration, factors that had strong effects on travel speeds included species, wild or hatchery-rearing history, watershed size and, in smaller rivers, body length. During the coastal migration, travel speeds were only strongly affected by species differences.

## Introduction

Migration is an important life history strategy in many animals, where individuals move among habitats at specific times of year or at specific ontogenetic stages to gain access to food resources, reduce predation risk, or find mates. Juveniles of most salmon species (*Oncorhynchus* spp. and *Salmo salar*) rear in freshwater and migrate to marine habitats to take advantage of better opportunities for growth; adults later migrate back to freshwater to spawn. Several costs are involved with migrations of salmon smolts: physiological changes for saltwater tolerance are metabolically costly, time spent migrating may in the short term take away from other possible uses of time such as feeding, and most importantly, smolts are vulnerable to predators along migration routes.

**Figure 1:**
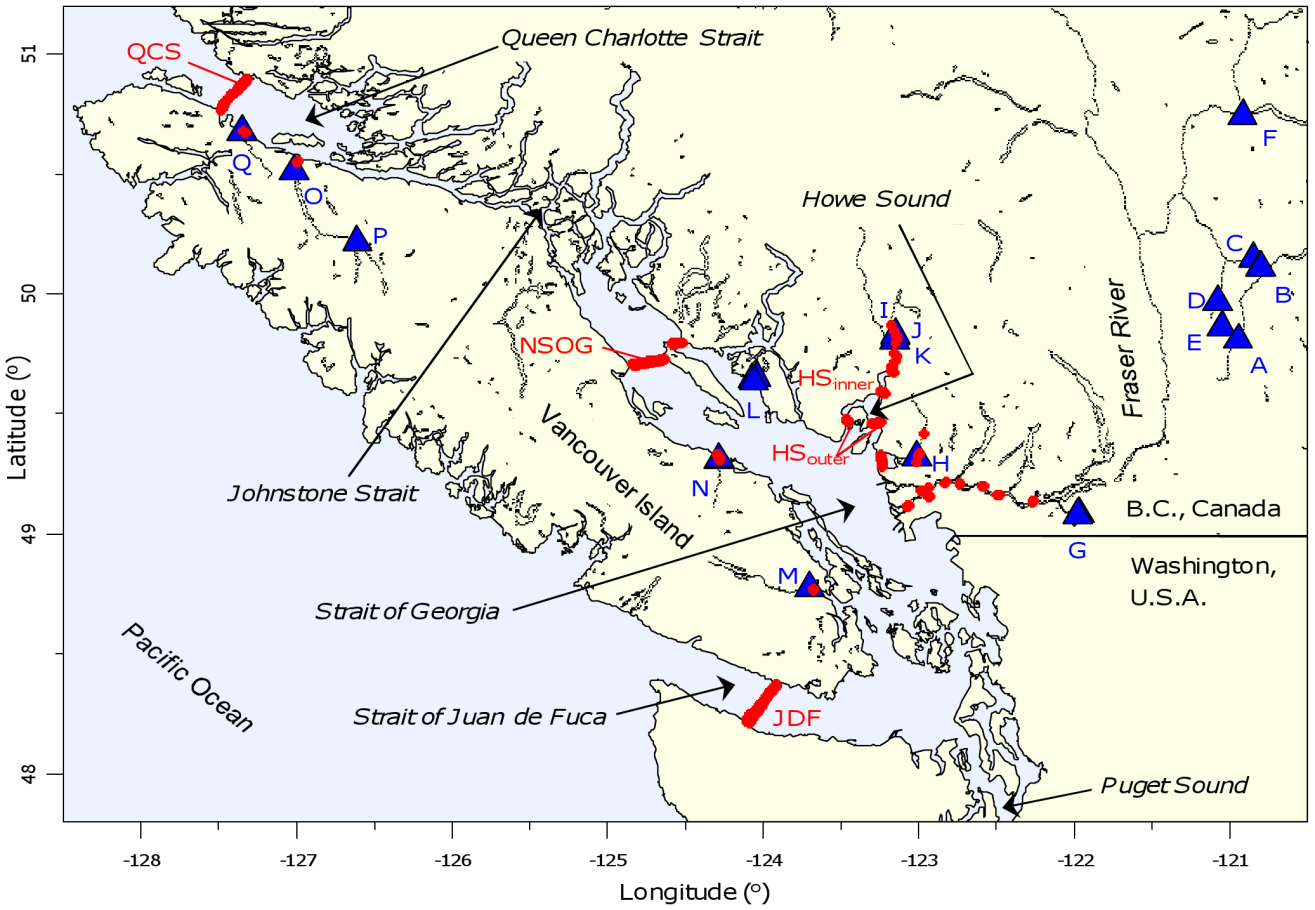
Map of study area in southern British Columbia. Red circles show acoustic receiver locations. Ocean receivers were in place for years 2004–2008 at Queen Charlotte Strait (QCS), northern Strait of Georgia (NSOG), Juan de Fuca Strait (JDF), inner Howe Sound (HS_inner_), and outer Howe Sound (HS_outer_). Not all receivers shown in rivers or at river mouths were in place every year. Not shown are receiver lines off the west coast of Vancouver Island, Washington, Oregon, or southeast Alaska, since B.C. smolts were rarely detected at these stations and analyses did not include them. Blue triangles show release locations of tagged salmon smolts. Release locations are labelled with letters corresponding to those in Table S1 (Mid-Fraser River sites: A–Coldwater River hatchery release site, B–Coldwater River rotary screw trap site, C–Nicola River hatchery release site, D–Spius Creek downstream hatchery release site, E–Spius Creek upstream hatchery release site, F–Deadman River rotary screw trap site. Lower Fraser River sites: G–Sweltzer Creek release site downstream from Cultus Lake. B.C. south coast sites: H–Seymour River hatchery release site, I–Cheakamus River upstream hatchery release site, J–Tenderfoot Creek hatchery release site, K–Cheakamus River downstream hatchery release sites and wild side channel trap sites, L–Sakinaw Lake. East coast Vancouver Island sites: M–Cowichan River hatchery release site, N–Englishman River rotary screw trap site, O–Nimpkish River rotary screw trap site and Gwa'ni Hatchery, P–Nimpkish River upstream release site (Woss Hatchery), Q–Keogh River fish fence).

Direction of travel after arrival in the ocean is potentially important to smolts: some migration routes are shorter than others (e.g., different routes around islands), and a shorter route may reduce time spent migrating and hence exposure to predators in coastal waters. Other factors may differ among possible directions as well, such as predator density and distribution, food availability, temperature, salinity, or strength of ocean currents. There generally appear to be species-specific migration routes through the inner coastal Strait of Georgia, British Columbia (B.C., Figure 1), with some species typically exiting the Strait northwards through Queen Charlotte Strait [1], [2], and other species typically southwards through Juan de Fuca Strait [3], with both routes leading to outer coastal waters. Within species, there appear to be some population-specific migration routes, even for populations that enter saltwater near the same location ([2]; similarly for Atlantic salmon [4]). Variation within populations may also exist, with some individuals moving in one direction and others moving in another. Such divergence in migration routes could be caused by various navigational cues (reviewed in [5]) or genetic factors. Salmon have been hypothesized to have two “zip codes” [6], with population-specific ocean feeding grounds in addition to their freshwater spawning grounds, and it is possible that similar variation in migration phenotypes could also occur within populations. Understanding direction of movement in juveniles may be particularly important in terms of fisheries management strategies if there is any correlation with direction taken during the return migration of adults.

Travel speed is also important to smolts, as faster speeds may also allow smolts to sooner escape from predation risk along riverine or coastal migration routes [7], where, compared with open pelagic habitats, predator densities may be higher or smolts may be more vulnerable due to the shallower and more constricted waterways. If predation risk is related to the time that smolts spend within rivers or coastal areas, then travelling faster through these habitats is expected to reduce exposure to predators.

In this paper, we present the most comprehensive analysis to date of juvenile salmon travel speeds and initial direction taken after ocean entry. We studied multiple populations of coho (*Oncorhynchus kisutch*), steelhead (*O. mykiss*), sockeye (or occasionally the supposedly freshwater variant, kokanee salmon, both *O. nerka*), and stream-type Chinook (*O. tshawytscha*) smolts during both downstream and inner coastal components of the migration. We quantified routes and travel speeds of individual fish by deploying a series of acoustic receivers in rivers and coastal areas. We consider migration patterns of salmon smolts that were tagged and released over five years (2004–2008) across a wide range of watersheds in southern B.C., under the Pacific Ocean Shelf Tracking Project (POST, see www.postcoml.org; [6]). All populations migrated within the confines of the Strait of Georgia system, bouned by Vancouver Island. We hypothesize several factors that may influence travel speeds of juvenile salmon: (i) species—coho and Chinook salmon often reside in coastal waters closer to their river of origin than steelhead and sockeye salmon [5], [8], so may reasonably be slower during their coastal migration; (ii) rearing history— hatchery-reared fish may require more time adjusting to river conditions after release, so may be slower to migrate downstream than wild fish; (iii) watershed of origin—smolts from large rivers may receive assistance from faster current velocities, so may travel downstream faster than smolts from smaller or slower rivers; and (iv) body size—larger fish are generally able to swim faster than smaller fish.

## Methods

### Ethics Statement

All work involving live fish reported in this paper was annually reviewed and approved as meeting or exceeding the standards laid out by the Canadian Council on Animal Care. Protocols were approved by the Pacific Region Animal Care Committee (Fisheries and Oceans Canada) in 2004 (# 04-026) and 2005 (# 05-004), and by the Animal Care Committee of Malaspina University-College (now Vancouver Island University) in 2006 (# 2006-08), 2007 (# 2006-08-R1), and 2008 (# 2006-08-R2).

### Field methods and study populations

A variety of VEMCO tag types were used for salmon smolts, mainly depending on fish size. All tags were individually-coded and transmitted pulse trains at 69 KHz. Most tags were either V7-2L (20 mm×7 mm diameter, 1.6 g in air, 136 dB re 1 µPa at 1 m) or V9-6L (21×9 mm, 2.9–3.1 g (depending upon year of manufacture), 142 dB) models, although for the larger hatchery-reared sockeye salmon smolts, occasionally larger tags were used, which permitted a longer battery life: V9-1L (24×9 mm; 3.6 g, 142 dB) or V9-2L (29×9 mm; 4.7 g, 142 dB) models. Most tags were programmed with a 30–90 s random time interval between successive transmissions of pulse trains (average 60 s). Acoustic tags were surgically implanted into smolts following standard protocols [9], [10]. Tagged smolts were generally held for ≥24 hr after surgery (up to several weeks for smolts tagged at hatcheries) to assess direct tagging-related mortality, tag extrusions, and monitor for signs of impaired swimming behaviour. These were rarely observed.

Acoustic receivers (69 kHz VEMCO VR-2 or VR-3 models) were used to detect acoustic tags. Receivers were deployed in successive locations along migration routes of salmon smolts to detect tagged fish during their migration out of freshwater and through the Strait of Georgia. Receivers were arranged in lines across the northern Strait of Georgia (NSOG), Juan de Fuca Strait (JDF), Queen Charlotte Strait (QCS), and two positions in Howe Sound (HS_inner_, HS_outer_) in 2004–2008 study years (Figure 1). Spacing between adjacent receivers in a line was generally 750–850 m apart, such that 4–31 receivers were required to span these inner coastal straits. This was predicted to provide sufficient overlap in detection radii of receivers (generally about 450 m in calm waters) that most tagged smolts are expected to transmit at least a few signals while crossing the line [6]. Receiver stations comprised of single or multiple receivers were deployed near the river mouths from which smolts emigrated (Figure 1; in the lower Fraser River, rather than right at the river mouth, 2–4 stations were deployed over ∼65 km of river length).

Salmon populations used in our analyses are listed in Table S1 of the Supporting Information section. Study populations consist of a group of smolts of a particular species from a particular watershed of origin with a particular provenance—either wild or hatchery-reared. Different smolt years typically consist of distinct cohorts of a population (e.g., fish that are 1.5 or 2.5 years old at smoltification), but in some populations ages of smolts may be mixed. Most study populations were tagged in 2004–2006, the main years of the POST Project demonstration phase (Table S1). The timing of migration was variable among smolt populations, even within the relatively narrow latitudinal range of southern B.C. Hatchery-reared fish were tagged and released at or near hatcheries, and wild fish were caught in river or side channel traps and held until they were tagged. Most smolts were released in May, but dates ranged from 15 April–1 July. Smolts were usually released at dusk to reduce the efficiency of visual predators immediately after release. Specific details about watersheds and salmon populations studied can be found in [11].

### Migration direction after ocean entry

The Strait of Georgia system framed by Vancouver Island and the B.C. mainland offers entry or exit points to the north (Queen Charlotte Strait) and to the south (Juan de Fuca Strait). Smolts entered the Strait of Georgia or Queen Charlotte Strait from several different locations—some to the north of Johnstone Strait, some from southern Vancouver Island, some from the Fraser River, and some from the south mainland coast, north of the Fraser River mouth (Figure 1). We estimated the proportion of fish from each release group listed in Table S1 that migrated northwards after ocean entry. Evidence of northward versus southward movement for fish from Keogh and Nimpkish Rivers, entering the ocean north of Johnstone Strait, was based on estimated numbers of fish crossing the QCS station (north) and the NSOG station (south). For all remaining populations that entered the Strait of Georgia south of NSOG, northward movement was based on the estimated number of fish crossing NSOG and southward movement was based on the estimated number of fish crossing the JDF station. These estimated numbers of fish were calculated simply as the number of fish detected at a station divided by the estimated detection probability for the station (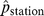) in the appropriate year and for the appropriate tag type [11]. Estimates 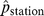 were year-specific and tag type-specific. These were estimated for non-terminal receiver stations (including NSOG) using Cormack-Jolly-Seber mark-recapture models fit to detection data of migrating salmon smolts in years 2004–2007. 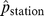 were predicted for terminal receiver stations (QCS and JDF) using the estimated 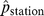 values from the NSOG station, and adjusting these for slight differences in the spacing between recovered receivers within a particular station in a particular year using multiple regression. Details are described in [11]. Within each release group, the proportion of fish migrating northward, P(*N*), was calculated as the estimated number crossing the northern line divided by the sum of the estimated numbers crossing northern and southern lines. Fish that migrated neither northward or southward (e.g., mortalities, residents) were not considered in the calculation, so P(*N*)+P(*S*) = 1.

For Fraser River and Cowichan River populations, distances from ocean entry to the northern station (NSOG) and southern station (JDF) were roughly equal (Figure 1). For other populations, the distance to the northern station was shorter than to the southern station. This was especially true for Sakinaw Lake and Englishman River populations, located only a short distance from NSOG, and for Keogh River and Nimpkish River populations, which were located close to QCS. If a fish was first detected at NSOG and later detected at JDF, only the last detection was considered in order to establish direction of migration (this rarely occurred).

### Travel speed analysis

Mean travel times of release groups were calculated for downstream (from release to detection near the river mouth) and coastal (from river mouth to ocean stations at NSOG, QCS or JDF) portions of the migration. These were measured as the time from release until the first detection of a tag at a river mouth or ocean station, and were averaged across fish within each population. For two of the 22 populations considered here (or six of the 49 release groups), travel speed estimates for some of the fish in these two populations have been previously published [12], [13], although they were aggregated differently for the coastal migration. Mean travel times were plotted against the minimum possible in-water migration distance (determined using Memory Map™ chart plotting software) for the appropriate portion of the migration to evaluate variation in average travel speed among groups. Coastal travel times were calculated as the difference between cumulative travel time from release to ocean lines and cumulative travel time from release to river mouth (rather than from only the subset of fish detected at both river mouths and ocean lines). Although not all fish were detected at river or ocean stations, we assume that travel speeds of fish that were detected are representative of fish not detected as well as the population of untagged fish.

Variation within release groups in travel speeds also exists, so most analyses focused on the level of individual fish. Travel speeds of salmon smolts were calculated for downstream and coastal portions of the migration, where coastal portions were from the river mouth to the terminal stations at QCS or JDF (so did not involve detections at NSOG). Two measures of travel speed were considered, which are commonly reported for fish speed: absolute rates in km·d^−1^ are presented here, while length-specific rates in BL·s^−1^ (body lengths per second) are presented in the Supporting Information section (Text S1). Absolute travel speeds were calculated as the minimum migration distance from release point to river mouth or from river mouth to terminal ocean station at QCS or JDF divided by the time elapsed between release and detection or subsequent detections. For the coastal portion, this required that a fish was detected at both the river mouth and at QCS or JDF. In total, travel speeds were assessed for 1,910 smolts spread among 42 release groups during the downstream migration, and for 487 smolts spread among 35 release groups for the coastal migration. Travel speed distributions were quantified, and were split among species and among wild or hatchery-rearing histories to assess possible differences among these grouping factors (sockeye and kokanee were grouped together for the coastal portion, as only 24 kokanee smolts were detected at QCS or JDF). (Kokanee are typically regarded to be the non-anadromous, freshwater-resident form of sockeye, although here fish confirmed by genetic samples to be kokanee (C. Wood, *pers. comm.*) were detected leaving the Strait of Georgia.)

Hierarchical linear mixed effects models were used to assess the effect on travel speeds of several possible explanatory factors, modelled as fixed effects: species (spp), wild or hatchery-rearing history (HW), fork length (FL), and whether fish were from a Fraser River or non-Fraser watershed (FnF). Sampling units were individual fish, but travel speeds are expected to be correlated within watersheds and years, especially during the downstream migration. A mixed-effects approach was therefore used, where individuals are related through their watershed of origin and year tagged. Watershed (nested within FnF) and year were considered as random effects, since travel speeds in particular years or of fish from particular watersheds were not of interest *per se*. These represented samples (although not strictly random samples) of a larger population of watersheds and years. Two datasets were analyzed independently, one for river travel speeds and the other for coastal travel speeds.

Several candidate models for travel speed were considered and compared using Bayesian Information Criterion (BIC; [14]) model selection methods. These candidate models represent competing hypotheses about which of the above factors were important in explaining variation in the travel speed data [15]; “importance” is assessed by the balance of goodness-of-fit of the model to the data and the number of model parameters required to achieve that fit. A global model was constructed assuming additivity of the above fixed effects, under the hypothesis that all factors had an important effect on travel speeds. For the river dataset, we also considered an interaction between FL and FnF as a fixed effect, which permitted different slopes of the relationship between travel speed and FL for these watershed categories. The reasoning behind this hypothesis is that river currents are faster in the Fraser River, so travel speeds are likely largely dominated by river flow. In other rivers with weaker flow, directed swimming is likely more important so body size may reasonably have a greater effect on travel speeds in the absence of strong currents. This interaction was not considered for the ocean dataset since fish from different watersheds all experience relatively low net flow conditions after ocean entry. The global model for travel speed, *u*, was specified as:
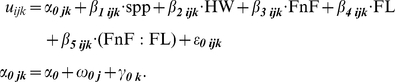
(1)


The term α_0 *jk*_ implies random intercepts for various levels of the random effects of watershed (ω_0 *j*_; nested within FnF) and year (γ_0 *k*_), where these random effects are normally distributed around zero, i.e., ω_0 *j*_∼*N*(0, 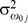) and γ_0 *k*_∼*N*(0, 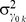). The residual error term is also assumed to be normally distributed after log-transformation of *u*, i.e., ε_0 *ijk*_∼*N*(0, 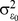). Model coefficients pair with dummy variables for spp, HW, and FnF, and represent slopes for FL and FnF:FL. In more abbreviated notation, we will represent this same model as:
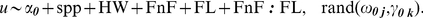
(2)


For each of the two datasets, several candidate models were compared as competing hypotheses. Given all the variables of interest, to avoid a very large number of candidate models, a two-step process was used. First, the full set of fixed effects from the global model was assumed for comparing random effects. Watershed nested within Fraser/non-Fraser, year, or both random effects were considered:

*u*∼(fixed), rand(ω_0 *j*_, γ_0 *k*_) Random intercepts for watersheds nested within Fraser/non-Fraser River, and years. Full set of fixed effects is assumed.

*u*∼(fixed), rand(ω_0 *j*_) Random intercepts for watersheds nested within Fraser/non-Fraser River. Full set of fixed effects is assumed.

*u*∼(fixed), rand(γ_0 *k*_) Random intercepts for years. Full set of fixed effects is assumed.

Second, the best set of random effects in terms of BIC was used to then compare models in terms of fixed effects [16]. Various reduced models were considered, again as multiple competing hypotheses. For the river dataset, these were:

*u*∼α_0_+spp+HW+FnF+FL+FnF:FL,rand(ω_0 *j*_, γ_0 *k*_) 

Full set of fixed effects on travel speed: species, rearing history, Fraser or non-Fraser origin, fork length, and a FnF:FL interaction. Random intercepts for watersheds nested within Fraser/non-Fraser River, and years.

*u*∼α_0_+spp+HW+FnF+FL,rand(ω_0 *j*_, γ_0 *k*_)

Fixed effects: species, rearing history, Fraser or non-Fraser origin, and fork length (no interaction). Random intercepts.

*u*∼α_0_+spp+HW+FnF,rand(ω_0 *j*_, γ_0 *k*_)

Fixed effects: species, rearing history, and Fraser or non-Fraser origin (no fork length or interaction). Random intercepts.

*u*∼α_0_+spp+FnF+FL,rand(ω_0 *j*_, γ_0 *k*_)

Fixed effects: species, Fraser or non-Fraser origin, and fork length (no rearing history or interaction). Random intercepts.

*u*∼α_0_+HW+FnF+FL,rand(ω_0 *j*_, γ_0 *k*_)

Fixed effects: rearing history, Fraser or non-Fraser origin, and fork length (no species or interaction). Random intercepts.

*u*∼α_0_+FnF+FL,rand(ω_0 *j*_, γ_0 *k*_) 

Fixed effects: Fraser or non-Fraser origin and fork length (no species, rearing history, or interaction). Random intercepts.

The list of candidate models used for the coastal dataset was the same, with one exception: an interaction was not considered between FnF:FL, as mentioned above, so model 1 in this second list was not considered. It was replaced by a model in which speeds after ocean entry were hypothesized to be common for otherwise-similar fish from the Fraser River and other rivers:

*u*∼α_0_+spp+HW+FL,rand(ω_0 *j*_, γ_0 *k*_)

Fixed effects: species, rearing history, and fork length (no Fraser or non-Fraser origin or interaction). Random intercepts.

Travel speeds were log-transformed prior to analyses since their distributions were typically log-normal (FL was also log-transformed to maintain the assumed linear relationship for conversion to BL·s^−1^). Comparison of models with differing random effects was done using a restricted maximum likelihood approach, while a maximum likelihood approach was used for comparing models with differing fixed effects [17].

## Results

Few coho (6.5% of tagged fish) or Chinook (1.9%) smolts from populations entering the Strait of Georgia were detected after entry to the main body of the Strait. These populations included hatchery-reared Tenderfoot Creek coho entering from Howe Sound as well as mostly hatchery-reared coho and Chinook from lower Thompson River tributaries (middle-Fraser River region; Table S1) entering the Strait from the Fraser River. In contrast, sockeye (28.5%) and steelhead (30.4%) smolts entering the Strait of Georgia were consistently detected migrating past ocean receiver stations as they left via either Johnstone and Queen Charlotte Straits to the north, or Juan de Fuca Strait to the south.

### Migration direction after ocean entry

**Figure 2:**
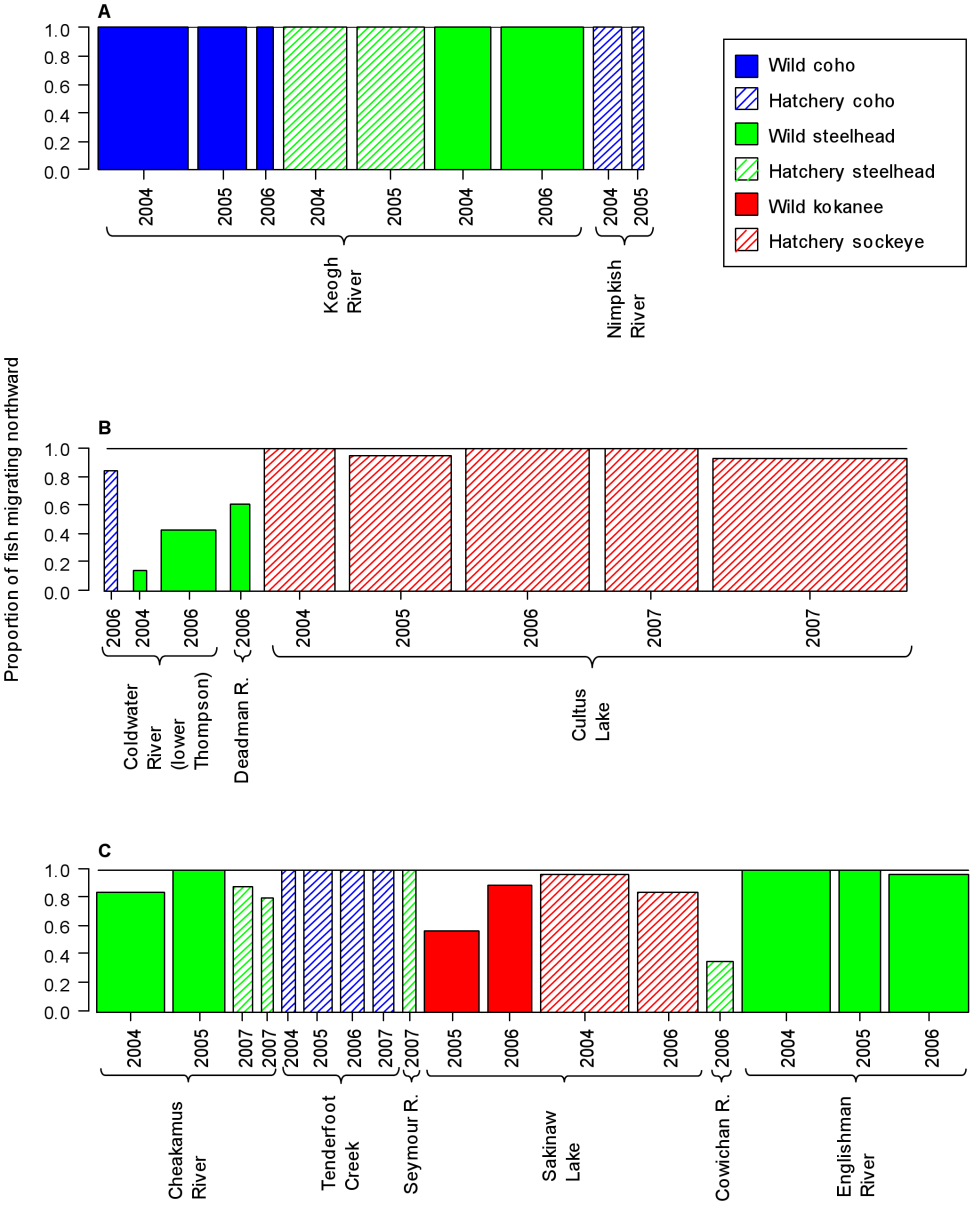
Proportion of fish migrating northward out of the Strait of Georgia system after ocean entry. Fish enter saltwater in Queen Charlotte Strait, north of the Strait of Georgia and Johnstone Strait (A), into the Strait of Georgia from the Fraser River (B), or into the Strait of Georgia from other rivers (C). In (A), direction is determined by detection at QCS (north) or NSOG (south). In (B) and (C), direction is determined by detection at NSOG (north) or JDF (south). All estimates are adjusted for station and year-specific detection probability estimates. Estimates are shown by species and wild or hatchery-rearing history. Bar width is proportional to the total number of fish detected at northern or southern stations; only populations with ≥5 detections in total are shown.

Within release groups, variation in direction of migration was observed in about one third of smolt populations, as some fish went north while others went south (Figure 2). About half of the populations entering into the Strait of Georgia had split migration route patterns. All populations entering Queen Charlotte Strait (Figure 2a) and the other half of populations entering the Strait of Georgia (Figure 2b, c) were only detected moving northwards. There were no consistent differences among species or rearing histories (wild versus hatchery) in P(*N*). There was some evidence of a latitudinal pattern, as expected, with populations entering the Strait of Georgia further to the north more likely to migrate northwards (Figure 1).

Few fish from mid-Fraser populations survived the downstream and coastal migrations. The four release groups that had ≥5 fish detected at either NSOG or QCS had variable P(*N*) estimates, ranging from <20% to >80%, but were based on only 6–23 fish detected (Figure 2b). In contrast, Cultus Lake sockeye from the lower Fraser moved almost exclusively northward, with only 8 of 249 detected fish taking the southern route across JDF.

Fish from other rivers adjacent to the Strait of Georgia showed a range of patterns in P(*N*). Most fish from south coast rivers (Cheakamus River, Tenderfoot Creek, Seymour River) migrated northwards, although some wild and some hatchery-reared Cheakamus River steelhead took the southern route (Figure 2c). Sakinaw Lake sockeye and kokanee also moved northward most often, but P(*N*) was slightly lower for wild kokanee than for the hatchery-reared sockeye. Englishman River steelhead moved northward almost exclusively, while the group from the Cowichan River, the only study river on southeastern Vancouver Island, moved predominantly southwards (Figure 2c).

### Travel speed analysis

**Figure 3:**
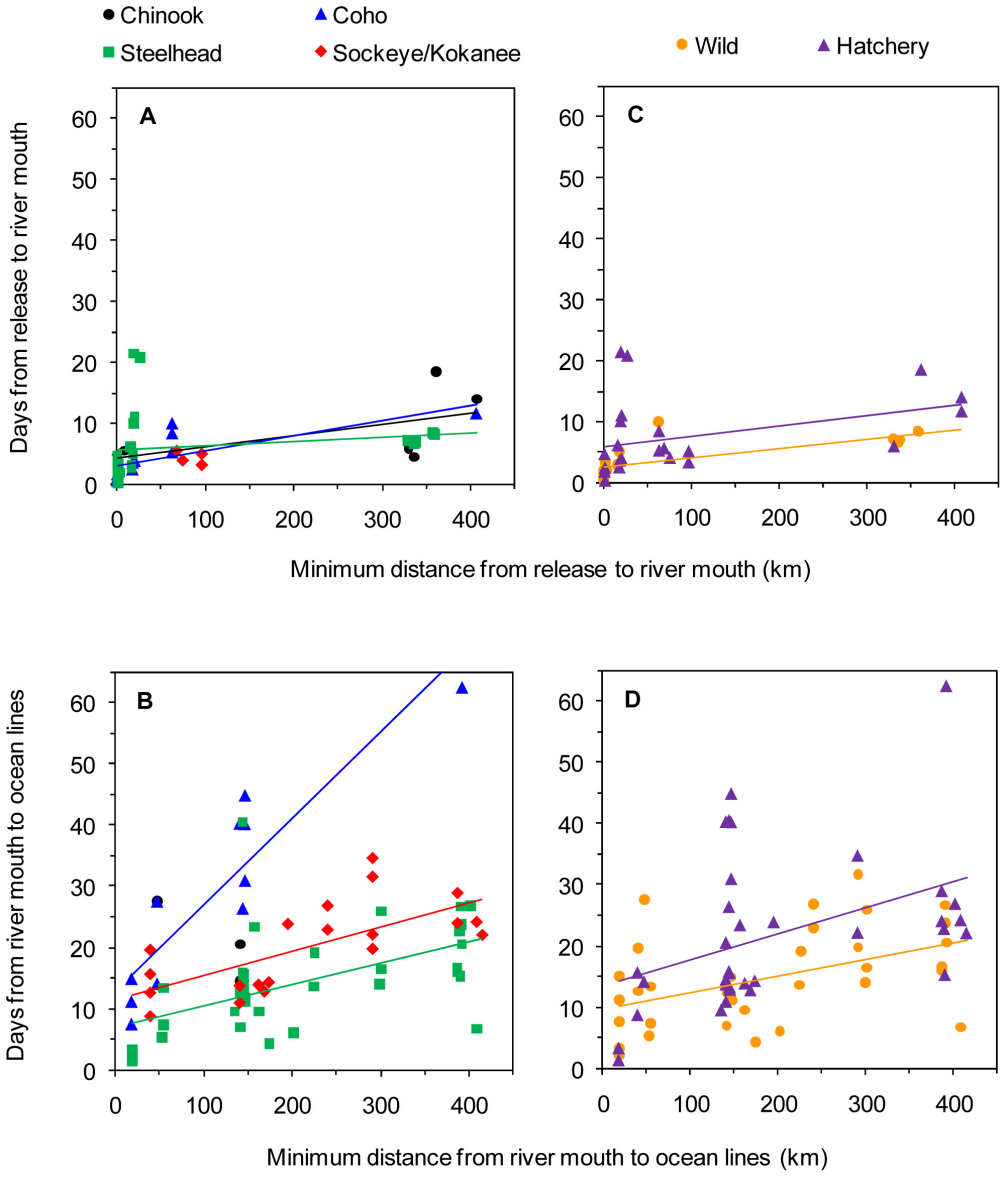
Mean travel time of smolt release groups as a function of minimum distance travelled. Travel time estimates are separated by species during the downstream (A) and coastal (B) migration, and separated by wild or hatchery-rearing history during the downstream (C) and coastal migration (D). Coastal travel times include times from ocean entry to the northern Strait of Georgia, Queen Charlotte Strait, or Juan de Fuca Strait lines, so each release group may have up to three points in (B) and (D). Lines show linear regressions fit to mean travel time point estimates for each species or rearing history separately.

Relationships between mean travel time of release groups and the minimum distance they migrated, either downstream or through the Strait of Georgia system, show among-group variability in travel speed. Tagged fish generally took <15 d on average to arrive at the river mouth after release (Figure 3a, c). Despite considerably longer distances travelled, mean travel times downstream were not much greater for mid-Fraser River populations (320–410 km) than for populations from other watersheds (<100 km). There was little difference among species in this pattern (Figure 3a), but hatchery-reared fish took about 5 more days to complete the downstream migration than wild fish, consistent across different migration distances (Figure 3c). Two outliers were hatchery-reared steelhead populations from the Cheakamus River in 2008 that took >20 d on average to migrate downstream despite distances of just 19 or 27 km. After ocean entry, species differences were observed in travel speeds. Coho populations took longer on average to reach the NSOG, QCS or JDF stations than steelhead or sockeye/kokanee populations for a similar migration distance (Figure 3b). Regression intercepts were >0 for all species, suggesting that even at relatively short distance from river mouth to ocean stations, smolts took more time on average to reach this distance than would be predicted if they migrated continuously after release. Hatchery fish also took more time on average to reach ocean stations after ocean entry than did wild fish, although variation in travel times was considerable for both rearing history groups (Figure 3d).

Variation in travel speeds also occurs among individuals within a given release group, which is not represented in Figure 3. Considering fish independently, travel speeds, *u*, ranged widely from near 0 to 189 km·d^−1^ (15.8 BL·s^−1^) during the downstream migration, and from near 0 to 48 km·d^−1^ (2.9 BL·s^−1^) during the coastal migration. Frequency distributions of travel speeds appeared to be approximately log-normal or exponential (Figure S1; ‘average’ travel speed implies the average speed during an individual's migration, not an average of individuals). Downstream travel speeds were much faster in the Fraser River (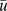 = 33.6 km·d^−1^, 2.40 BL·s^−1^) than in other rivers (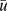 = 6.6 km·d^−1^, 0.50 BL·s^−1^), and the outlying individuals with speeds of >100 km·d^−1^ tended to be from the Fraser River. Travel speeds during the coastal migration were intermediate between these (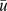 = 12.8 km·d^−1^, 0.82 BL·s^−1^). Recall that travel speeds are calculated assuming the minimum distance from release to river mouth or river mouth to outer ocean lines, so if actual fish migration routes were longer than this shortest path, actual speeds would be faster.

**Figure 4:**
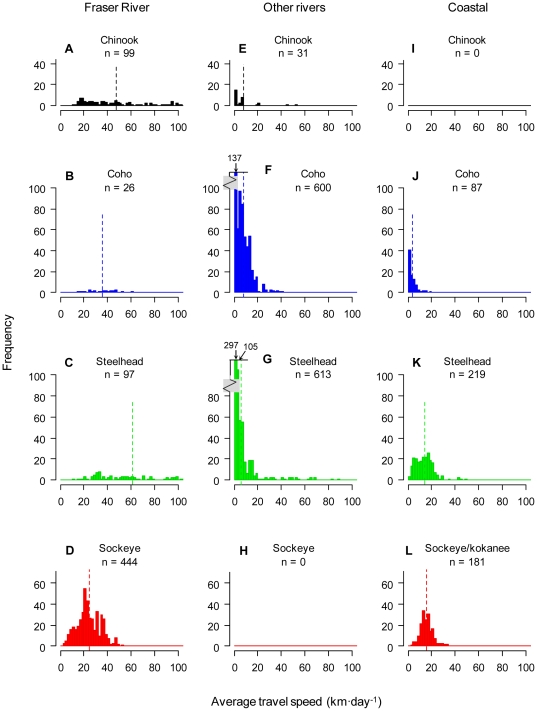
Histograms of travel speeds during the downstream and coastal migrations, separated by species. Panels A–D show speeds down the Fraser River, panels E–H show speeds down other rivers, and panels I–L show speeds during the coastal migration. Frequency distributions are truncated at 100 km·d^−1^. Number of fish is indicated for each category. Dashed lines show the mean travel speed of each species and habitat combination. Note the axis break in panels F and G.

**Figure 5:**
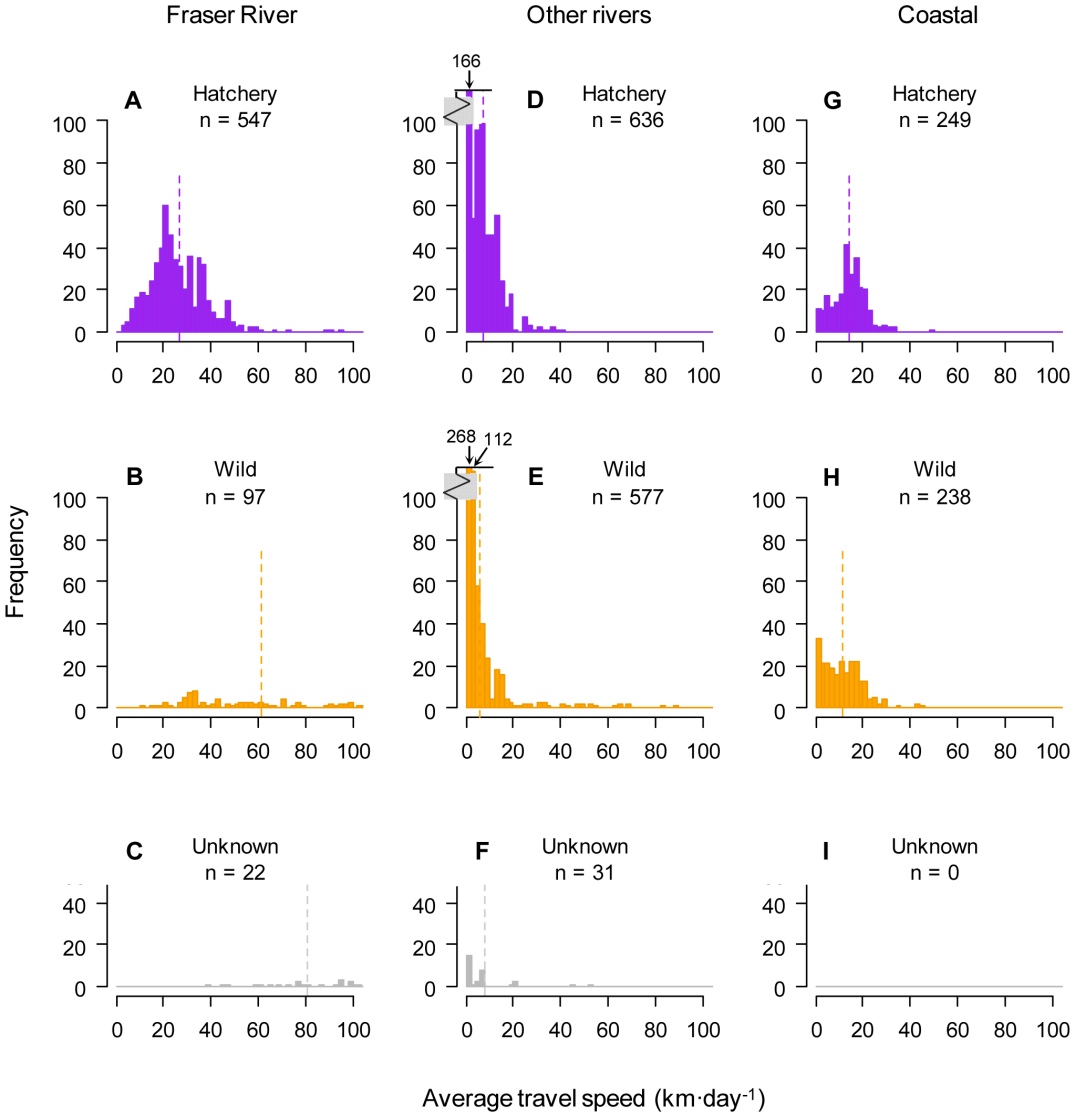
Histograms of travel speeds during the downstream and coastal migrations, separated by rearing history. Panels A–C show speeds down the Fraser River, panels D–F show speeds down other rivers, and panels G–I show speeds during the coastal migration. All species are included. Frequency distributions are truncated at 100 km·d^−1^. Number of fish is indicated for each category. Dashed lines show the mean travel speed of each rearing history and habitat combination. Note the axis break in panels D and E.

In rivers other than the Fraser River, one characteristic that stands out is the large proportion of fish that moved <2 km·d^−1^ during the downstream migration (Figure S1). Separating the data by species, steelhead appeared to move slower than coho (Figure 4). These slow-moving steelhead were of both wild and hatchery origin (Figure 5; note that mainly steelhead are represented by this slowest-moving category). Across all species, travel speeds during the downstream migration in rivers other than the Fraser were generally similar between wild and hatchery-reared fish (Figure 5). In the Fraser River, Cultus Lake sockeye (from the lower Fraser) had slower travel speeds during the downstream migration than populations of Chinook, coho, or steelhead from the mid-Fraser River (Figure 4). This population difference (Cultus Lake sockeye are hatchery-reared) was the main reason for an observed difference in downstream travel speeds between wild and hatchery fish in the Fraser River (Figure 5). During the coastal migration, the coho smolts that were detected at both river mouth and QCS/JDF stations moved at a slower net speed between these stations (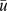 = 3.8 km·d^−1^, 0.29 BL·s^−1^) than did steelhead (*u* ^ = 13.9 km·d^−1^, 0.88 BL·s^−1^) or sockeye/kokanee (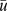 = 15.9 km·d^−1^, 1.00 BL·s^−1^) smolts (Figure 4). Travel speeds of wild and hatchery-reared smolts were generally similar after ocean entry (Figure 5).

**Figure 6:**
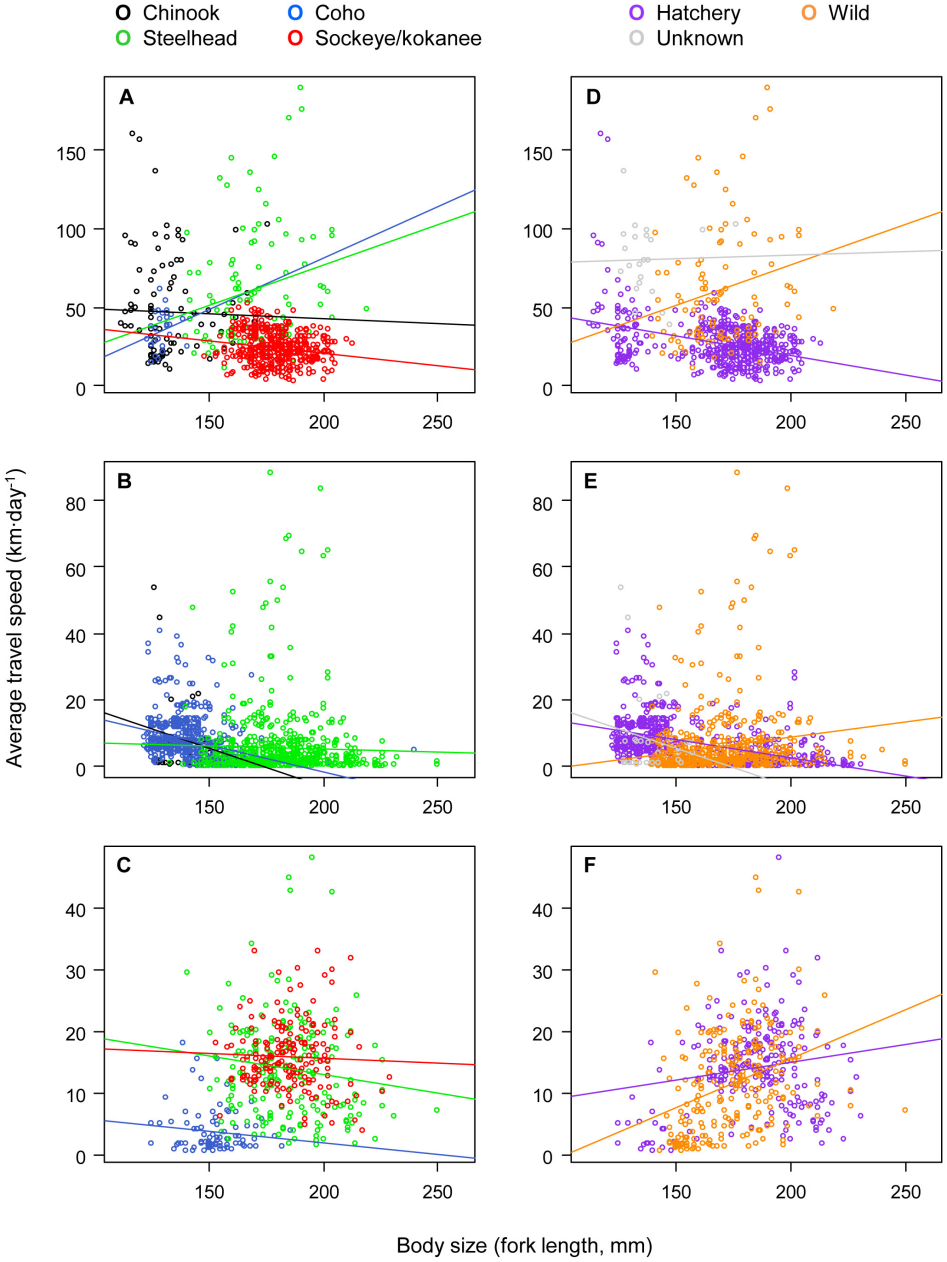
Average absolute travel speeds vs. body length at time of tagging. Panels A–C show travel speed estimates separated by species, while panels D–F show estimates separated by rearing history. All estimates are in units of km·d^−1^. Data points represent individual fish, and are separated for downstream (Fraser River: A, D; other rivers: B, E) and coastal (C, F) portions of the migration. Lines show linear regressions fit to travel speeds for each species or rearing history separately. Note the different scales on the travel time axes for Fraser River, other river, and coastal segments.

Relationships between travel speed and body length were generally weak or inconsistent in direction. In the Fraser River, positive relationships between absolute travel speeds and body length were observed for coho and steelhead as well as for wild fish in general (Figure 6a,d), but slight negative relationship were seen for sockeye and for hatchery fish in general (Figure 6a,d). In other rivers, travel speed vs. body length relationships were weak when separated by species (Figure 6b) and weak but in opposite directions for wild and hatchery-reared fish (Figure 6e). During the coastal migration there was relatively little effect of fork length on average absolute travel speeds (Figure 6c,f). Similar patterns were observed for length-adjusted travel speeds (Figure S2).

In comparisons of mixed-effects candidate models for variation in travel speed, the best set of random effects included both watershed nested within Fraser/non-Fraser rivers and year. This was true for both river and coastal datasets (Table 1). Of these two effects, ω_0 *j*_ was the stronger in terms of explaining variation in *u*, especially for downstream travel speeds, as expected. These random effects were both included in candidate models for comparing fixed effects as competing hypotheses. Similar model selection results were observed when considering length-adjusted travel speed instead of absolute speed (Table S2).[Table pone-0012916-t002]

**Table 1:** Model selection results for comparison of random effects in models for absolute travel speeds.

Model^a^	*k*	−2·ln(*L*)	BIC	ΔBIC
**Downstream travel speeds**				
*u*∼(fixed), rand(ω_0 *j*_, γ_0 *k*_)	13	5037.5	5135.7	0.0
*u*∼(fixed), rand(ω_0 *j*_)	12	5107.2	5197.8	62.1
*u*∼(fixed), rand(γ_0 *k*_)	11	5838.6	5921.7	786.0
**Coastal travel speeds**				
*u*∼(fixed), rand(ω_0 *j*_, γ_0 *k*_)	10	778.3	840.2	0.0
*u*∼(fixed), rand(ω_0 *j*_)	9	815.7	871.4	31.2
*u*∼(fixed), rand(γ_0 *k*_)	8	824.7	874.2	34.0

Comparison criteria include number of parameters (*k*), negative log-likelihood (−2·ln(*L*)), and the Bayesian Information Criterion (BIC).

a In all models, fixed parameters included additive effects of species, wild or hatchery-rearing history, Fraser River or non-Fraser origin (FnF), and fork length (FL). For the rivers dataset, a FnF:FL interaction was also included. There was also one fewer species group (Chinook) and one fewer rearing history group (‘unknown’) in the coastal dataset compared with the rivers dataset, hence three fewer fixed parameters overall in the coastal dataset. Models differ in their random effects considered: (γ_0 *k*_), random intercepts for years; (ω_0 *j*_), random intercepts for watersheds nested within Fraser/non-Fraser River; (ω_0 *j*_, γ_0 *k*_), random intercepts for both effects.

**Table 2 pone-0012916-t002:** Model selection results for comparison of fixed effects in models for absolute travel speeds.

Model^a^	*k*	−2·ln(*L*)	BIC	ΔBIC
**Downstream travel speeds**				
*u*∼α_0_+spp+HW+FnF+FL+FnF:FL	13	5029.0	**5127.2**	**0.0**
*u*∼α_0_+spp+HW+FnF+FL	12	5056.7	5147.4	20.2
*u*∼α_0_+spp+HW+FnF	11	5088.5	5171.6	44.4
*u*∼α_0_+HW+FnF+FL	9	5121.0	5189.0	61.8
*u*∼α_0_+spp+FnF+FL	10	5132.0	5207.5	80.3
*u*∼α_0_+FnF+FL	7	5231.0	5283.9	156.6
**Coastal travel speeds**				
*u*∼α_0_+spp+HW+FnF	9	767.8	**823.5**	**0.0**
*u*∼α_0_+spp+FnF+FL	9	768.2	823.9	0.4
*u*∼α_0_+spp+HW+FL	9	770.6	826.3	2.8
*u*∼α_0_+spp+HW+FnF+FL	10	767.8	829.7	6.2
*u*∼α_0_+FnF+FL	7	914.2	957.5	134.0
*u*∼α_0_+HW+FnF+FL	8	911.6	961.1	137.6

Comparison criteria include number of parameters (*k*), negative log-likelihood (−2·ln(*L*)), and BIC. The lowest BIC value in each comparison is boldfaced.

a In all models, random effect parameters consisted of random intercepts for watersheds nested within Fraser/non-Fraser River and random intercepts for years, i.e., rand(ω_0 *j*_, γ_0 *k*_).

In terms of fixed effects, ranking of candidate models differed among river and coastal datasets. For downstream travel speeds, the strongest supported hypothesis by far was the global model (Table 2; this was also the case for length-adjusted speeds, Table S3). Species and rearing history differences were therefore both observed after accounting for body size variation and river size (Fraser vs. non-Fraser). Steelhead travel speeds during the downstream migration were slower on average than those of other species (confidence limits around the estimated coefficient excluded zero; *β*_1, steelhead_ = −0.43; 95% c.l., −0.83 to −0.03, compared to the Chinook reference group). There was some indication that coho were faster than the other species, all else equal (*β*_1, coho_ = 0.38; 95% c.l., −0.03 to 0.79). Wild fish migrated faster downstream than hatchery-reared fish, all else equal (*β*_2, wild_ = 0.80; 95% c.l., 0.65–0.96, compared to the hatchery group). There was a large overall difference in *u* between Fraser River fish and smolts from other rivers, with greater *u* downstream in the Fraser River (*β*_3, Fraser_ = 17.0; 95% c.l., 11.5–22.5), although this overall difference was dependent on an interaction with body length. The slope of ln(*u*) versus ln(FL) was not different from zero in the Fraser River (*β*_4, Fraser:FL_ = −0.32; 95% c.l., −1.22 to 0.58), but was positive for fish from other rivers (*β*_5, nF:FL_ = 2.86; 95% c.l., 1.78–3.94).

For absolute travel speeds during the coastal migration, the strongest supported hypothesis was the model that included predictor variables of species, wild or hatchery-rearing history, and Fraser or non-Fraser origin, but did *not* include body length (Table 2). A model involving additive effects of spp, FnF, and FL, but not HW, had nearly the same level of support (ΔBIC = 0.4; this was also the best-supported model for length-adjusted speeds, Table S3). A third model involving additive effects of spp, HW, and FL (but not FnF) also had a considerable amount of support within the dataset (ΔBIC = 2.8). We look further at the fit of the top model to absolute travel speed estimates. Multiple regression results confirm the slower travel speeds of coho during the coastal migration compared to those of steelhead and sockeye (Figure 4; *β*_1, steelhead_ = 1.34, 95% c.l., 1.17–1.51; *β*_1, sockeye_ = 1.29, 95% c.l., 0.73–1.84, compared to the coho reference group). Despite being factors in the best model, neither HW nor FnF had confidence limits that excluded zero (*β*_2, wild_ = 0.05, 95% c.l., −0.09 to 0.20, compared to the hatchery group; *β*_3, Fraser_ = −0.45, 95% c.l., −0.94 to 0.05, compared to the non-Fraser group). A further reduced model was not part of the original model set, but in a post-hoc comparison, the model [*u*∼α_0_+spp, rand(ω_0 *j*_, γ_0 *k*_)] outperformed all others in the original model set (*k* = 7, −2·ln(*L*) = 771.2, BIC = 814.5). Thus, it appears that species is the only fixed effect that explains a sufficient amount of variation in coastal travel speeds to warrant inclusion as a parameter in the model.

## Discussion

Animal migration behaviours are incredibly diverse at the species level in terms of distance, timing, and routes of travel. Within species, variation also exists among and even within populations, as we have documented here for juvenile salmon in terms of the initial direction of migration and travel speeds. There is growing interest in quantifying variation in migration strategies among individuals [18]; describing the extent of individual variation in such strategies is a necessary first step for quantifying the ecological and evolutionary significance of this variation.

### Migration direction after ocean entry

The proportion of fish within a release group migrating northwards after ocean entry ranged widely among groups. Fish entering saltwater in the northern Strait of Georgia or Queen Charlotte Strait moved predominantly northwards, while those entering further to the south displayed split migration routes. Even further south, steelhead from five Hood Canal populations (Puget Sound, Washington State) were only detected leaving via JDF [19]. While previous studies have documented variation in migration direction through the Strait of Georgia among species or populations [2], [3], we found intra-population variation among individuals, in three different species. Previous studies have suggested that ocean currents may play a role in the net direction travelled by salmon smolts [1], [8]. There is no clear effect of ocean currents on directional patterns in our study; reversing tidal streams are stronger in the southern Strait of Georgia than in the northern portion, but are bi-directional along both exit routes [20]. Net run-off from the Fraser River (accounting for approximately 75% of freshwater run-off into the Strait of Georgia) moves predominantly out of the Strait of Juan de Fuca, although complex and variable circulation patterns within the Strait [20] are such that the direction and strength of surface currents which smolts face at any particular time are unknown.

In some cases the estimated P(*N*) was likely biased due to unequal distances to the northern and southern stations. Since higher total mortality is expected for longer migration distances (assuming a constant mortality rate per unit distance), P(*N*) is biased upwards for the populations whose route to the northern station is shorter than the route to the southern station. This is especially true for Sakinaw Lake and Englishman River populations, as the distance to NSOG was much shorter than the distance to JDF (Figure 1). To note, if P(*N*) were calculated based on detections at QCS and JDF instead of NSOG and JDF, then for these two populations the five estimates of P(*N*)≥90% would change very little, but the P(*N*) estimates for 2005 Sakinaw Lake kokanee and 2006 Sakinaw Lake sockeye would decrease from 56% and 84% to 23% and 60%, respectively. In contrast, the bias in P(*N*) would be negligible for Fraser and Cowichan River populations, as distances to northern and southern stations were roughly equal. Bias in P(*N*) could be reduced by incorporating it directly into mark-recapture models as an estimated parameter and treating survival separately along northern and southern routes [21]. For populations with relatively few fish detected at either northern or southern stations, however, such methods are prohibitive if P(*N*) tends to vary among populations, as they appear to.

Biases in estimates of P(*N*) can also be caused by incorrect assumed values for detection probabilities at ocean detection stations. While 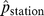 can be reliably estimated for NSOG using mark-recapture methods, these had to be extrapolated for predictions at terminal stations QCS and JDF ([11]; alternatives to extrapolation include using only local detection information at terminal stations, [22]). Further, these estimates or predictions of 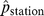 are year-, station-, and tag type-specific (adjusting for variation in the spacing between recovered receivers), but they represent averages of all smolts in a migration year. Several environmental factors may cause detection probabilities to vary over hourly or daily temporal scales (reviewed in [23] and Melnychuk, *in review*), and this fine-scale variation is not captured in estimates of 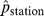. Estimated numbers of fish crossing a particular station vary inversely with 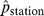, so overestimates of 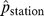 would lead to underestimates of the number of smolts crossing the station, and vice-versa. We did not detect any consistent trends in 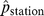 at ocean stations during the migration season (at the scale of weeks or months; [11]), however, so our population-level estimates of P(*N*) are likely reasonable as they represent averaged quantities across smolts and across the migration season.

As previously mentioned, very few coho smolts entering the main body of the Strait of Georgia were detected moving across terminal stations at QCS or JDF, which may have been due to summer residency in the Strait rather than high mortality during a directed migration. The high P(*N*) of coho populations (100% for the Tenderfoot Creek release groups and 84% for the Coldwater River group) are based almost exclusively on fish detections at NSOG without subsequent detection at terminal stations. Only five Strait of Georgia coho (from Tenderfoot Creek in 2006 and 2007) were detected at QCS, and only one (a Coldwater River coho in 2006) was detected at JDF. It is possible that fish were foraging near NSOG and detected, rather than migrating continuously northward, so these estimates of P(*N*) might not actually represent directed migration behaviour. Tag batteries implanted into coho smolts prior to their downstream migration last only a few months, so if smolts resided in the Strait of Georgia over the summer and left during the fall or winter, tag batteries would have likely expired thus fish would not be detected at QCS or JDF. In a related study, however, marine-resident coho post-smolts were trawl-caught, tagged, and released in the Strait of Georgia in July and September of 2006 so that possible out-migration behaviour after the first ocean summer could be assessed [24]. Of all the coho post-smolts detected leaving the Strait of Georgia system (59 of 173 originally tagged), only 4 fish were detected at QCS, with the other 55 (93%) detected at JDF. The frequency with which coho post-smolts took the southern route increased over time, being low and similar to the proportion taking the northern route from July–September, but increasing thereafter throughout winter months [24]. It is also possible that some coho and Chinook do not leave the Strait of Georgia at all. Earlier studies have suggested that some coho spend their entire marine life in the Strait, and the proportion of a population residing may vary annually with salinity [25], [26]. Differences among salmon species in duration of residence in the Strait of Georgia likely reflect diet differences, as coho and Chinook are less reliant on small invertebrate prey by June (compared to sockeye, pink, and chum salmon) [27], [28]. Small invertebrate prey abundance declines after May, so other species may not satisfy food requirements if they were to reside in the Strait like coho and Chinook.

Similar arguments of a possible lack of migration out of the Strait of Georgia may apply to some smolts from Sakinaw Lake kokanee and sockeye release groups. It appears that some fish from these groups have non-migratory behaviours, either remaining in the Strait throughout their ocean lives or returning to Sakinaw Lake. About 20–30% of smolts from 2004 hatchery sockeye and 2005 wild kokanee groups were detected at QCS or JDF stations, but none of these fish were detected thereafter as returning adults [29] (these fish had ‘sleeper’ tags that turned on again during the return migration). In contrast, of the 57% of fish from the 2004 group that were not detected at any station as smolts, 5% of these were detected as adults near the release site (similarly, 7% for the 2005 group). Since these fish were detected neither leaving nor returning via Queen Charlotte or Juan de Fuca Straits, this suggests that some fish did not leave the Strait of Georgia [29]. There were also other indications of non-migratory behaviour, as about 12% of the wild kokanee smolts released in 2006 were detected entering Sakinaw Lake several days after release directly into the ocean, and remaining there [29].

It is not clear whether the direction of migration in juveniles correlates with that of returning adults. Previous studies showed this is not necessarily the case for Fraser River sockeye taking the northern or southern route around Vancouver Island [1]. The direction taken by returning adults has varied under different ocean regimes, with most returning through Juan de Fuca Strait during 1953–1977, and most returning through Queen Charlotte Strait since then [30], [31]. Population-specific migration routes [2], [3], [4], [32], [33] and rearing or foraging grounds (e.g., adult coho [34]; and juvenile Chinook , R.J. Beamish, *pers. comm.*) have been observed in some fish meta-populations. Further, direction of migration of returning adult Fraser River sockeye salmon may be predictable from brain physiology measured during the return migration near the Queen Charlotte Islands, prior to arrival at a bifurcation point north of Vancouver Island (K. Miller-Saunders, *pers. comm.*). Adult sockeye retained the differentiated brain profiles throughout the remainder of their migration up the Fraser River. If direction of travel may be at least in part deterministic several days or weeks ahead of the point where a fish takes the northern or southern route, then it is possible this has a genetic component, in which case the direction taken by smolts may also in part be deterministic. If smolt outmigration and adult return migration route directions are correlated, then quantifying the direction taken by juveniles may help to inform management decisions about area and time fishery closures during the adult return migration. For example, Harrison Lake sockeye salmon (a Fraser River sub-population) tend to take the southern exit route out of Juan de Fuca Strait and also have relatively high marine survival rates compared with most other Fraser sub-populations that typically take the northern exit route [2]. Threatened or at-risk populations that tend to take one particular return route (at a particular time) could be allowed to escape the fishery through closures, allowing fisheries to operate along other migration routes that may not involve as many weak stocks.

Similar to split-route migration paths within populations, recent evidence shows that within coho [35], Chinook [3] and sockeye [2] populations along the outer coast from Oregon to southeast Alaska, fish fall into discrete groups with respect to migration speeds. In coho, there is a fast, northward-migrating component and a slow component in which fish delay migrating until winter and then move northwards more slowly. Coho populations originating from the Strait of Georgia and Puget Sound predominantly have the ‘slow’ strategy, with only 3.5% of populations having a fast-migrating component, and with some populations overwintering off the west coast of Vancouver Island rather than migrating further northward [35]. This is apparent on a regional scale as well as on a population scale, and is line with the observations of at least some coho delaying outmigration from the Strait of Georgia until winter months [24], [27].

### Travel speeds during downstream and coastal migration

Observed travel speeds were variable, but generally within the range of those observed in other studies (reviewed in [36]; for two of the 22 populations considered here, some of the travel speed data have been published previously [12], [13]). Species differences in travel speeds were observed, but the relationship differed between river and coastal portions of the migration. In rivers, steelhead had slower average travel speeds than other species. This was likely a result of temporary delaying of migrations rather than slower travel while actually migrating. For example, two hatchery-reared steelhead groups from the Cheakamus River in 2008 took a relatively long time on average to migrate a fairly short distance downstream (Figure 3a; across years, the four hatchery-reared release groups from the Cheakamus River took on average over three times as long to migrate downstream than the three wild release groups, two of which were reported in [12]). Nearly half of all steelhead smolts across watersheds had very slow average migration speeds of <2 km·d^−1^, which was a much higher proportion than those seen in other species (Figure 4). Slow migration speeds were also observed for wild and hatchery-reared steelhead from Hood Canal (3.1 km·d^−1^; [19]). In the Columbia River, in contrast to sockeye and Chinook smolts, an inverse relationship between body length and downstream migration speed was observed for steelhead [37]. Such migratory behaviour differences between steelhead and other species may relate to the propensity for freshwater residualization, i.e., failure to migrate to the ocean [38]. Steelhead are closely related genetically to the non-anadromous rainbow trout (and are, in fact, the same species) [39]. In a meta-analysis across several watersheds, on average about 2–9% of hatchery steelhead smolts from a given cohort have been found to residualize (S. Hausch and M. Melnychuk, *unpubl. data*), either delaying migration for an additional year of freshwater rearing, or permanently, essentially adopting a rainbow trout life history strategy. There may also be a period of delayed migration in steelhead smolts prior to the start of seaward migration, which would explain the slower average migration speeds downstream.

In rivers, coho were slightly faster on average than other species after accounting for other factors such as rearing history, Fraser or non-Fraser watershed, and fork length. After ocean entry, however, coho moved slower on average than steelhead or sockeye smolts. Since coastal travel speeds were defined as from river mouth to exit from the Strait of Georgia via QCS or JDF, most coastal travel speed data for coho came from Keogh and Nimpkish River populations, which entered Queen Charlotte Strait rather than the Strait of Georgia proper (as previously mentioned, only a few coho from other populations were detected at QCS or JDF. In contrast, most steelhead and sockeye/kokanee smolts detected were from populations that entered the Strait of Georgia directly.) In part, coho travel speeds during the coastal migration were slower simply because of the shorter migration distance for most fish, and any ‘distance-independent’ components of coastal travel time being averaged over a shorter total travel time (see below). The few Strait of Georgia coho detected at terminal stations, however, did have greater average travel times than steelhead and sockeye that migrated similar distances (Figure 3), so shorter distances cannot fully explain the slower average coastal travel speeds of coho smolts. As mentioned above, coho and Chinook smolts entering the Strait of Georgia were rarely detected, and it is not clear whether this is primarily due to high mortality after ocean entry or summer residency in the Strait away from detection stations, such as in the Gulf Islands or Fraser plume regions [27]. The fall and winter emigrations of coho post-smolts out of the Strait, primarily via Juan de Fuca Strait [24], suggest that at least part of the pattern is due to summer residency. The few coho smolts from Strait of Georgia populations detected at QCS or JDF may have spent some time foraging in the Strait before emigration, thus leading to slower average travel speeds than steelhead or sockeye/kokanee, which tended to emigrate from the Strait soon after ocean entry (this broad analysis confirms earlier, population-specific results for steelhead and sockeye; [12], [13]). The observed differences among species in travel speeds are not surprising given previous observations on species differences in foraging behaviour [27].

Smolts from the Fraser River generally had much faster travel speeds downstream than smolts from other rivers, which is largely explained by faster river flow. River velocity is also likely the reason why Chinook, coho and steelhead from the mid-Fraser had faster travel speeds than Cultus Lake sockeye, which were released near the lower Fraser where the river is wide and currents are consequently slower. Travel speed differences between fish from the Fraser River and those from other rivers is also partly explained by differential proportions of time spent not migrating. Smolts in smaller rivers were rarely detected during daylight hours [11], and this diurnal holding behaviour slowed their average speed of downstream migration. This is likely the main reason why downstream migration speeds in these other rivers were slower on average than coastal speeds, even though coastal travel would have little assistance from currents. In contrast, Atlantic salmon smolts were observed to travel faster during the day than at night in a coastal bay [40].

The higher flows in the Fraser compared to other rivers might also explain why body length had little effect on downstream travel speed in the Fraser River, but had a positive effect in other rivers, with large individuals migrating faster on average. Fish body length may have little effect on travel speeds if river flows contribute substantially to movement downstream (in contrast to coastal migrations, where most movement results from directed swimming). Movement down the Fraser River may be largely passive, whereas more active movement downstream might be required in other rivers to complete the downstream migration quickly. Larger individuals are generally better able to swim faster. Surprisingly, this hypothesis was not supported during the coastal migration; large and small individuals migrated from the river mouth to QCS or JDF lines at similar average absolute travel speeds.

Travel speed differences were observed between wild and hatchery-reared fish during the downstream migration, with wild fish moving faster than hatchery-reared fish on average. It appears from Figure 3c that this difference is attributed not to slower speeds in hatchery fish while travelling, but to an intercept difference in travel time versus distance, i.e., a distance-independent factor. Even at very short distances downstream, hatchery fish took about 5 d more to complete the downstream migration, and this difference was consistent across the range of downstream distances studied. This difference could simply be due to hatchery-reared smolts waiting several days before initiating seaward migration, whereas wild fish were caught while actively migrating. Reasons for hatchery fish delaying their migration could include: seeking refuge while stress levels slowly decrease after release, since creek or river environments are substantially different than hatchery environments; holding in waters near the release site to imprint on the local water (imprinting is common during smoltification and aids in finding the watershed of origin when adults later return to spawn); or holding somewhere during the downstream migration until smoltification is complete, in the case of hatchery release groups that had forced rather than volitional release strategies used. After ocean entry, wild and hatchery-reared smolts migrated at similar average absolute travel speeds.

Similar to the downstream migration, intercepts >0 in a travel time–distance relationship for the coastal migration were also observed for coho and sockeye/kokanee (Figure 3). Although a simple linear regression through point estimates for steelhead also had an intercept >0, the fit was not convincing as the travel times for migration distances of <20 km were short, suggestive of an actual non-linear intercept close to 0 for these steelhead groups. The intercept of about 10 d for sockeye/kokanee is largely driven by smolts from Sakinaw Lake detected at the NSOG station, 40 km from the release site. These smolts were mostly released directly into the ocean near the lake outlet, so it is reasonable that a saltwater acclimation period could have been necessary before actively migrating. Many Cultus Lake sockeye and Sakinaw Lake smolts were also detected entering Howe Sound (Figure 1) prior to leaving via either QCS or JDF [13], [29], so this 10-day intercept may have also resulted from time spent searching for migration route cues or even foraging periods shortly after ocean entry. Healey [27] documented similar migration times of sockeye through the Strait of Georgia, although he found that most sockeye took the Juan de Fuca Strait exit. The difference may relate to watershed of origin, as most sockeye caught were likely from wild up-river Fraser populations in contrast to the lower-river hatchery smolts used in this study. The intercept for coho was also about 10 d, and this was driven by smolts entering Queen Charlotte Strait from the Keogh and Nimpkish Rivers. This could have resulted from similar explanations of osmotic adjustment, initial estuarine or marine foraging, or migration route-seeking shortly after ocean entry. In other studies, coho smolts have been shown to temporarily reside in estuaries between 1–5 d on average before continuing their migration [41], [42]. An alternative explanation for these populations is partial summer residency in Queen Charlotte Strait. Coho smolts may have spent several days after ocean entry milling or foraging, after which a portion of fish migrated northwards across QCS and another portion resided for the summer. This possibility is admittedly speculative, but consistent with the observation that apparent survival to QCS was generally lower for Nimpkish and Keogh River coho populations than for Keogh River steelhead populations despite similar migration distances [11] (low apparent survival can be interpreted not only as mortality but also as residency without possibility of detection).

It is not clear how travel speeds along a migration route affect the risk of predation. If predation risk is proportional to distance travelled (which may occur for single encounters between a migrating smolt and an ambush predator [43]), then a smolt is subject to the same risk whether it travels slowly or rapidly. If predation risk is instead proportional to the time spent within rivers or coastal areas (which may occur if predators are highly mobile and repeated encounters with predators are expected [43]), or is somewhere between these extremes, then travelling faster through coastal waters is expected to reduce exposure to predators. Although some seal [44] or fish (e.g., bull trout or Dolly Varden char) predators in rivers may be ambush predators, many smolt predators like birds [45], [46], seals [47], and dogfish [48] are highly mobile and may encounter a particular smolt repeatedly. Future work should rigorously quantify the relationship between travel speed and mortality of migrating smolts.

In summary, we documented considerable diversity in migration behaviours among salmon smolts of different species, rearing histories, and watersheds; migration patterns and the factors affecting them varied across different habitats. During the downstream migration, the factors that had the greatest effect on travel speeds were species, wild or hatchery-rearing history, Fraser or non-Fraser origin, and, in smaller rivers, body length. During the coastal migration, only the species factor explained a large amount of variation in travel speeds. Although tagged smolts could only be detected at acoustic receiver stations, these migration patterns are expected to collectively represent the outcome of a complex balance between foraging and predator avoidance requirements. Migration strategies likely have direct fitness consequences through variation in exposure to predation risk; future studies should assess the direct impact of such migration patterns on survival probabilities of migrating salmon smolts.

## Supporting Information

Text S1 This contains two sections, in addition to a references section: Populations and previously-published data, Travel speeds in body lengths per second.
(0.04 MB DOC)

Table S1 Southern British Columbia salmon smolt populations tagged under POST from 2004–2008.
(0.11 MB DOC)

Table S2 Model selection results for comparison of random effects in models for length-adjusted travel speeds.
(0.04 MB DOC)

Table S3 Model selection results for comparison of fixed effects in models for length-adjusted travel speeds.
(0.04 MB DOC)

Figure S1 Histograms of travel speeds of tagged fish during the downstream and coastal migrations under alternate travel speed measures. Panels A–D show absolute speeds, while panels E–H show length-adjusted speeds. Frequency distributions are truncated at 100 km⋅d^−1^ and 10 BL⋅s^−1^, as few fish had speeds faster than these. Number of fish is indicated for each category. Dashed red lines show the mean travel speed in each habitat for each measure.
(0.26 MB TIF)

Figure S2 Average length-adjusted travel speeds vs. body length at time of tagging. Panels A–C show travel speed estimates separated by species, while panels D–F show estimates separated by rearing history. All estimates are in units of BL⋅s^−1^. Data points represent individual fish, and are separated for downstream (Fraser River: A, D; other rivers: B, E) and coastal (C, F) portions of the migration. Lines show linear regressions fit to travel speeds for each species or rearing history separately. Note the different scales on the travel time axes for Fraser River, other river, and ocean segments.
(0.62 MB TIF)
