# Supplementary material for: Spatio-Temporal Migration Patterns of Pacific Salmon Smolts in Rivers and Coastal Marine Waters
Source: PLoS One. 2010 Sep 23;5(9):e12916. doi: 10.1371/journal.pone.0012916 (PMC2944838; doi:10.1371/journal.pone.0012916)
Supplement: Text S1 — This contains two sections, in addition to a references section: Populations and previously-published data, Travel speeds in body lengths per second. (0.04 MB DOC) [file pone.0012916.s001.doc]

# Supporting Information Text

## Populations and previously-published data

Table S1 lists the salmon smolt populations that were considered for our analyses. Note that some of the travel speed data for two of these 22 populations (or six of the 49 release groups) are previously published. Cultus Lake sockeye travel speeds for 2004-2006 and some of the 2007 fish were given in [1]. Cheakamus River steelhead travel speeds for 2004-2005 were given in [2]. For both populations, previously-published travel speeds were aggregated differently for the early ocean migration, and new data are considered in the present manuscript.

## Travel speeds in body lengths per second

Absolute travel speeds in km·d^‑1^ are provided in the main text. Length-specific rates in body lengths per second (BL·s^‑1^, which were fork lengths measured at time of tagging) are presented here. It is expected that large fish travel faster than small fish and that a conversion from average speed in km·d^‑1^ to average speed in BL·s^‑1^ corrects for this expected difference among individuals. The conversion often over-corrects the relationship, since length-specific speeds optimal for cruising generally vary inversely with fish length [3,4]. A similar analysis for length-specific travel speeds was conducted as for absolute travel speeds.

Figure S1 shows travel speeds during the downstream migration of the Fraser River and other rivers, and during the coastal migration. Note the shapes of the histograms are similar between the two measures of travel speed. Histograms of travel speeds in BL·s^‑1^ when separated out by species or by rearing history are not shown here, but also have similar shapes to those of absolute speeds in km·d^‑1^ shown in Figures 4 and 5, respectively.

Under the common assumption that larger fish travel faster than smaller fish, fish travel speeds are often converted from absolute measures (km·d^‑1^) to length-specific measures (BL·s^‑1^). This conversion is typically assumed to correct for length-dependent variation in travel speeds, such that fish of different lengths will have resulting speeds in BL·s^‑1^ that are more similar. If little variation exists among individuals of different body sizes in average absolute travel speeds, however, this conversion may over-correct, resulting in faster length-specific travel speeds in smaller fish. This may be the case for some salmon smolts when grouped by species. For example, during the coastal migration there was relatively little effect of fork length on average absolute travel speeds (Figure 6). When adjusted for body length, however, travel speeds in BL·s^‑1^ decreased as fork length increased (Figure S2). This pattern may simply result from the particular range of fork lengths within each species, however, because when fish were instead separated by wild or hatchery rearing history, the BL·s^‑1^ measure properly corrected the positive relationship between absolute speed and length for hatchery fish, and partly corrected it for wild fish (Figure S2). Since length-adjusted measures are often of interest for quantifying travel speeds in salmon smolts, we also consider measures of BL·s^‑1^ in a multiple regression analysis.

Similar to absolute speed (Table 1), the best set of random effects for variation in length-adjusted travel speed included both watershed nested within Fraser/non-Fraser rivers and year (Table S2). Note that the results for these random effects comparisons for length-adjusted travel speeds (Table S2) are similar to those for absolute travel speeds (Table 1) because fork length is incorporated among the fixed effects for both measures.

In terms of fixed effects, the best model for length-adjusted travel speeds during the downstream migration was the global model (Table S3), similar to the case for absolute travel speeds. Since the conversion from km·d^‑1^ to BL·s^‑1^ is a simple linear transformation, all models involving FL were identical in terms of likelihood and BIC between the two measures (Tables 2 and S3; the only differences between measures lie in the estimates for intercept and slope coefficients for FL). The single model not involving FL was closer to the best model for the BL·s^‑1^ measure (ΔBIC = 16.9, Table S3) than for the km·d^‑1^ measure (ΔBIC = 44.4; Table 2), simply indicating that the conversion to BL·s^‑1^ in the response variable did improve the model fit if FL was not explicitly included as a predictor variable.

For the coastal migration, the best model for length-adjusted travel speeds differed from the best model for absolute travel speeds. The top candidate model for the length-adjusted measure of *u* involved additive effects of species, body length, and Fraser or non-Fraser origin, but did *not* include wild or hatchery-rearing history (Table S3; this model also had considerable support for absolute travel speeds, as shown in Table 1). A second model with a considerable level of support involved additive effects of species, body length, and wild or hatchery-rearing history, but not Fraser or non-Fraser origin (ΔBIC = 2.3, Table S3).

# Supporting Information References

1. Welch DW, Melnychuk MC, Rechisky ER, Porter AD, Jacobs MC, et al. (2009) Freshwater and marine migration and survival of endangered Cultus Lake sockeye salmon smolts using POST, a large-scale acoustic telemetry array. Canadian Journal of Fisheries and Aquatic Sciences 66: 736-750.

2. Melnychuk MC, Welch DW, Walters CJ, Christensen V (2007) Riverine and early ocean migration and mortality patterns of juvenile steelhead trout (*Oncorhynchus mykiss*) from the Cheakamus River, British Columbia. Hydrobiologia 582: 55-65.

3. Bellwood DR, Fisher R (2001) Relative swimming speeds in reef fish larvae. Marine Ecology-Progress Series 211: 299-303.

4. Trudel M, Welch DW (2005) Modeling the oxygen consumption rates in Pacific salmon and steelhead: Model development. Transactions of the American Fisheries Society 134: 1542-1561.
