# Supplementary material for: Spatio-Temporal Migration Patterns of Pacific Salmon Smolts in Rivers and Coastal Marine Waters
Source: PLoS One. 2010 Sep 23;5(9):e12916. doi: 10.1371/journal.pone.0012916 (PMC2944838; doi:10.1371/journal.pone.0012916)
Supplement: Table S1 — Southern British Columbia salmon smolt populations tagged under POST from 2004–2008. (0.11 MB DOC) [file pone.0012916.s002.doc]

**Table S1.** Southern British Columbia salmon smolt populations tagged under POST from 2004–2008.

|  | Watershed | Species^a^ | H/W^b^ | Year | *n*^c^ | FL ± SD^d^  (Range) | Release date(s)^e^  (*n*) | Tag type(s)  (*n*) | FW dist^f^ | Map label^g^ |
| --- | --- | --- | --- | --- | --- | --- | --- | --- | --- | --- |
| **Middle Fraser River** | | |  |  |  |  |  |  |  |  |
|  | Coldwater River | Chnk | H | 2006 | 100 | 151.2 ± 17.1  (125 – 186) | Apr 18 13:15 | V7-2L | 407.1 | A |
|  | Coldwater River | Chnk | UN | 2005 | 69 | 139.3 ± 11.2  (121 – 177) | May 04 20:00 (58), May 08 20:00 (11) | V7-2L (50), V9-6L (19) | 336.2 | B |
|  | Coldwater River | Coho | H | 2004 | 40 | 130.8 ± 2.7  (127 – 141) | May 31 14:00 | V7-2L (12), V9-6L (28) | 349.1 | A |
|  | Coldwater River | Coho | H | 2006 | 100 | 130.1 ± 3.3  (125 – 141) | May 29 13:00 | V7-2L | 407.1 | A |
|  | Coldwater River | Sth | W | 2004 | 31 | 154.4 ± 18.9  (125 – 204) | Apr 16 23:00 (2), May 07 20:00 (4), May 15 20:00 (16), May 31 21:15 (9) | V9-6L | 329.7 | B |
|  | Coldwater River | Sth | W | 2005 | 50 | 170.6 ± 15.4  (142 – 219) | Apr 20 20:00 (1), May 04 20:00 (11), May 08 20:00 (4), May 19 20:00 (17), May 29 21:30 (17) | V9-6L | 336.2 | B |
|  | Coldwater River | Sth | W | 2006 | 75 | 162.3 ± 13.1  (140 – 198) | May 07 20:00 (56), May 18 20:00 (19) | V7-2L (25), V9-6L (50) | 347.1 | B |
|  | Nicola River | Chnk | H | 2004 | 49 | 129.4 ± 4.8  (120 – 142) | Apr 21 10:00 | V9-6L | 323.5 | C |
|  | Nicola River | Chnk | H | 2005 | 50 | 117.1 ± 3.1  (112 – 127) | May 02 15:39 | V7-2L | 330.0 | C |
|  | Spius Creek | Chnk | H | 2006 | 99 | 128.6 ± 4.4  (123 – 142) | Apr 20 10:35 | V7-2L | 360.9 | D |
|  | Spius Creek | Coho | H | 2005 | 50 | 128.4 ± 3.9  (122 – 141) | May 19 11:15 | V7-2L | 355.6 | E |
|  | Deadman River | Sth | W | 2005 | 57 | 166.5 ± 18.8  (142 – 227) | Apr 20 22:15 (9), Apr 27 21:30 (8), May 05 23:30 (11), May 17 21:45 (6), May 30 21:15 (23) | V9-6L | 337.7 | F |
|  | Deadman River | Sth | W | 2006 | 64 | 163.2 ± 20.5  (128 – 209) | May 08 12:00 (3), May 19 22:00 (12), May 28 22:00 (10), Jun 10 22:00 (12), Jun 19 21:30 (22), Jul 01 21:30 (5) | V7-2L (26), V9-6L (38) | 359.1 | F |
| **Lower Fraser River** | | |  |  |  |  |  |  |  |  |
|  | Cultus Lake | Sock | H | 2004 | 100 | 177.6 ± 12.5  (142 – 210) | May 03 20:30 | V9-6L | 68.6 | G |
|  | Cultus Lake | Sock | H | 2005 | 376 | 176.8 ± 13.8  (148 – 216) | Jun 08 20:00 | V9–1L (92), V9-2L (188), V9-6L (96), | 75.1 | G |
|  | Cultus Lake | Sock | H | 2006 | 200 | 178.3 ± 8.9  (164 – 204) | Apr 20 18:00 | V9–1L | 96.5 | G |
|  | Cultus Lake | Sock | H | 2007 | 119 | 171.4 ± 5.9  (160 – 185) | Apr 26 15:00 | V9-6L | 96.5 | G |
|  | Cultus Lake | Sock | H | 2007 | 200 | 188.7 ± 8.2  (170 – 217) | May 16 15:00 | V9–1L | 96.5 | G |
| **South coast** | | | |  |  |  |  |  |  |  |
|  | Cheakamus River | Sth | H  (1) | 2007 | 19 | 186.4 ± 9.6  (172 – 206) | May 06 16:00 | V9-6L | 20.3 | J |
|  | Cheakamus River | Sth | H  (2) | 2007 | 81 | 182.6 ± 11.6  (163 – 210) | May 23 12:30 | V9-6L | 15.9 | K |
|  | Cheakamus River | Sth | H  (1) | 2008 | 40 | 176.7 ± 8.7  (158 – 192) | May 05 16:00 | V9-6L |  | J |
|  | Cheakamus River | Sth | H  (2) | 2008 | 58 | 185.1 ± 12.7  (155 – 206) | May 08 11:17 (40), May 22 09:37 (18) | V9-6L |  | I, K |
|  | Cheakamus River | Sth | W | 2004 | 51 | 184.5 ± 17.3  (148 – 226) | May 08 20:45 (42), May 24 18:00 (9) | V9-6L | 16.9 | K |
|  | Cheakamus River | Sth | W | 2005 | 49 | 177.6 ± 14  (153 – 212) | May 06 20:25 (44), May 19 20:20 (5) | V9-6L | 16.6 | K |
|  | Cheakamus River | Sth | W | 2008 | 100 | 177.9 ± 11.9  (149 – 209) | May 06 19:30 (72), May 12 20:30 (28) | V9-6L |  | K |
|  | Tenderfoot Creek | Coho | H | 2004 | 100 | 140.2 ± 6.6  (130 – 159) | May 10 18:00 | V9-6L | 18.0 | J |
|  | Tenderfoot Creek | Coho | H | 2005 | 100 | 138.1 ± 7.3  (123 – 155) | May 11 15:00 | V7-2L (50), V9-6L (50) | 17.7 | J |
|  | Tenderfoot Creek | Coho | H | 2006 | 120 | 138.9 ± 9.9  (125 – 165) | May 04 16:00 | V7-2L (70), V9-6L (50) | 17.8 | J |
|  | Tenderfoot Creek | Coho | H | 2007 | 199 | 132.2 ± 5.5  (125 – 147) | May 10 16:00 | V7-2L | 20.3 | J |
|  | Sakinaw Lake | Kok | W | 2005 | 47 | 191.3 ± 12.3  (171 – 213) | Jun 02 12:00 | V9–1L | NA | L |
|  | Sakinaw Lake | Kok | W | 2006 | 74 | 198.9 ± 13.3  (175 – 224) | Jun 01 11:20 (25), Jun 02 21:15 (49) | V9–1L (72), V9-6L (2) | NA | L |
|  | Sakinaw Lake | Sock | H | 2004 | 97 | 193 ± 14.6  (172 – 251) | Jun 01 11:30 (63), Jun 16 14:00 (34) | V9–1L | NA | L |
|  | Sakinaw Lake | Sock | H | 2006 | 62 | 213.4 ± 11.5  (170 – 239) | Jun 02 21:15 | V9–1L (50), V9-6L (12) | NA | L |
|  | Seymour River | Sth | H | 2007 | 60 | 185.9 ± 12.6  (160 – 224) | Apr 27 15:15 (20), May 08 14:50 (20), May 15 12:00 (20) | V9-6L | 1.2 | H |
| **East coast Vancouver Island** | | | |  |  |  |  |  |  |  |
|  | Cowichan River | Sth | H | 2006 | 50 | 177.8 ± 13.8  (156 – 200) | May 09 15:10 | V9-6L | 1.6 | M |
|  | Englishman River | Sth | W | 2004 | 67 | 174.4 ± 16.9  (132 – 210) | May 13 22:00 (12), May 14 22:30 (7), May 15 22:30 (12), May 17 21:40 (12), May 18 21:40 (9), May 19 21:30 (4), May 20 21:30 (6), May 21 22:00 (1), May 22 22:10 (2), May 23 22:00 (2) | V9-6L | 3.8 | N |
|  | Englishman River | Sth | W | 2005 | 43 | 159.4 ± 14.8  (133 – 196) | May 13 21:00 (11), May 18 21:30 (12), May 19 19:00 (14), May 23 11:30 (6) | V9-6L | 2.3 | N |
|  | Englishman River | Sth | W | 2006 | 50 | 168.7 ± 12.5  (149 – 206) | May 03 17:00 (8), May 04 21:00 (6), May 05 21:00 (16), May 07 21:00 (20) | V9-6L | 1.6 | N |
|  | Keogh River | Coho | W | 2004 | 107 | 154.6 ± 6.8  (139 – 173) | May 27 09:30 (37), May 27 22:00 (40), May 28 22:00 (30) | V9-6L | 0.8 | Q |
|  | Keogh River | Coho | W | 2005 | 49 | 157.5 ± 10.5  (143 – 183) | May 01 19:30 | V9-6L | 0.8 | Q |
|  | Keogh River | Coho | W | 2006 | 50 | 152.4 ± 18.6  (140 – 240) | May 07 19:30 (20), May 10 21:00 (30) | V9-6L | 0.8 | Q |
|  | Keogh River | Sth | H | 2004 | 92 | 188 ± 18.4  (150 – 231) | May 17 16:00 (50), May 19 11:00 (42) | V9-6L | 0.8 | Q |
|  | Keogh River | Sth | H | 2005 | 50 | 204 ± 14.7  (163 – 247) | May 25 16:00 | V9-6L | 0.8 | Q |
|  | Keogh River | Sth | W | 2004 | 78 | 174.6 ± 20  (147 – 250) | May 27 09:30 (25), May 27 22:00 (8), May 28 22:00 (13), May 29 22:00 (9), Jun 04 22:00 (23) | V9-6L | 0.8 | Q |
|  | Keogh River | Sth | W | 2006 | 50 | 184 ± 17.1  (159 – 232) | May 31 19:30 | V9-6L | 0.8 | Q |
|  | Nimpkish River | Chnk | UN | 2006 | 50 | 139 ± 9.6  (126 – 171) | May 08 19:00 | V7-2L | 8.3 | O |
|  | Nimpkish River | Coho | H | 2004 | 99 | 140.6 ± 4.2  (125 – 151) | Apr 28 18:00 (2), Jun 14 15:30 (97) | V9-6L | 61.7 | P |
|  | Nimpkish River | Coho | H | 2005 | 57 | 130.9 ± 7  (122 – 149) | Jun 17 16:00 | V7-2L (49), V9-6L (8) | 62.8 | P |
|  | Nimpkish River | Coho | W | 2006 | 50 | 145.9 ± 4.8  (140 – 165) | May 11 16:20 | V9-6L | 62.8 | P |

^a^ Species abbreviations: Chnk, Chinook; Kok, kokanee; Sock; sockeye; Sth, steelhead.

^b^ Rearing history abbreviations: H, hatchery-reared; W, wild-caught without an adipose fin clipped so presumed wild; UN, unknown provenance since wild-caught but with unclipped hatchery fish released upstream.

^c^ Number of smolts tagged and released.

^d^ Mean fork length, in mm, ± 1 standard deviation. Range of FL in parentheses.

^e^ Release times are Pacific daylight savings times.

^f^ Distance from release point to river mouth (or lowest in-river receiver), in km.

^g^ Letters in the last column correspond to release locations on the map in Figure 1.
