# Supplementary material for: Spatio-Temporal Migration Patterns of Pacific Salmon Smolts in Rivers and Coastal Marine Waters
Source: PLoS One. 2010 Sep 23;5(9):e12916. doi: 10.1371/journal.pone.0012916 (PMC2944838; doi:10.1371/journal.pone.0012916)
Supplement: Table S2 — Model selection results for comparison of random effects in models for length-adjusted travel speeds. (0.04 MB DOC) [file pone.0012916.s003.doc]

**Table S2.** Model selection results for comparison of random effects in models for length-adjusted travel speeds.

| Model ^a^ | *k* | –2·ln(*L*) | BIC | | ∆BIC |
| --- | --- | --- | --- | --- | --- |
| **Downstream travel speeds** |  |  |  | |  |
| *u*~(fixed), rand(ω_0_ *_j_*, γ_0_ *_k_*) | 13 | 5037.5 | | 5135.7 | 0.0 |
| *u*~(fixed), rand(ω_0_ *_j_*) | 12 | 5107.2 | | 5197.8 | 62.1 |
| *u*~(fixed), rand(γ_0_ *_k_*) | 11 | 5838.6 | | 5921.7 | 786.0 |
| **Coastal travel speeds** |  |  | |  |  |
| *u*~(fixed), rand(ω_0_ *_j_*, γ_0_ *_k_*) | 10 | 778.3 | | 840.2 | 0.0 |
| *u*~(fixed), rand(ω_0_ *_j_*) | 9 | 815.7 | | 871.4 | 31.2 |
| *u*~(fixed), rand(γ_0_ *_k_*) | 8 | 824.7 | | 874.2 | 34.0 |

Comparison criteria include number of parameters (*k*), negative log-likelihood (–2·ln(*L*)), and the Bayesian Information Criterion (BIC).

^a^ In all models, fixed parameters included additive effects of species, wild or hatchery-rearing history, Fraser River or non-Fraser origin (FnF), and fork length (FL). For the rivers dataset, a FnF**:**FL interaction was also included. There was also one fewer species group (Chinook) and one fewer rearing history group (‘unknown’) in the coastal dataset compared with the rivers dataset, hence three fewer fixed parameters overall in the coastal dataset. Models differ in their random effects considered: (γ_0_*_k_*), random intercepts for years; (ω_0_ *_j_*), random intercepts for watersheds nested within Fraser/non-Fraser River; (ω_0_ *_j_*, γ_0_ *_k_*), random intercepts for both effects.
