# Supplementary material for: Spatio-Temporal Migration Patterns of Pacific Salmon Smolts in Rivers and Coastal Marine Waters
Source: PLoS One. 2010 Sep 23;5(9):e12916. doi: 10.1371/journal.pone.0012916 (PMC2944838; doi:10.1371/journal.pone.0012916)
Supplement: Table S3 — Model selection results for comparison of fixed effects in models for length-adjusted travel speeds. (0.04 MB DOC) [file pone.0012916.s004.doc]

**Table S3.** Model selection results for comparison of fixed effects in models for length-adjusted travel speeds.

| Model ^a^ | *k* | –2·ln(*L*) | BIC | ∆BIC |
| --- | --- | --- | --- | --- |
| **Downstream travel speeds** |  |  |  |  |
| *u*~ α_0_+spp+HW+FnF+FL+FnF**:**FL | 13 | 5029.0 | **5127.2** | **0.0** |
| *u*~ α_0_+spp+HW+FnF | 11 | 5061.0 | 5144.1 | 16.9 |
| *u*~ α_0_+spp+HW+FnF+FL | 12 | 5056.7 | 5147.4 | 20.2 |
| *u*~ α_0_+HW+FnF+FL | 9 | 5121.0 | 5189.0 | 61.8 |
| *u*~ α_0_+spp+FnF+FL | 10 | 5132.0 | 5207.5 | 80.3 |
| *u*~ α_0_+FnF+FL | 7 | 5231.0 | 5283.9 | 156.6 |
| **Coastal travel speeds** |  |  |  |  |
| *u*~ α_0_+spp+FnF+FL | 9 | 768.2 | **823.9** | **0.0** |
| *u*~ α_0_+spp+HW+FL | 9 | 770.6 | 826.3 | 2.3 |
| *u*~ α_0_+spp+HW+FnF+FL | 10 | 767.8 | 829.7 | 5.7 |
| *u*~ α_0_+spp+HW+FnF | 9 | 777.3 | 833.0 | 9.0 |
| *u*~ α_0_+FnF+FL | 7 | 914.2 | 957.5 | 133.6 |
| *u*~ α_0_+HW+FnF+FL | 8 | 911.6 | 961.1 | 137.1 |

Comparison criteria include number of parameters (*k*), negative log-likelihood (–2·ln(*L*)), and BIC. The lowest BIC value in each comparison is boldfaced.

^a^ In all models, random effect parameters consisted of random intercepts for watersheds nested within Fraser/non-Fraser River and random intercepts for years, i.e., rand(ω_0_ *_j_*, γ_0_ *_k_*).
