# Supplementary figures and images for: Spatio-Temporal Migration Patterns of Pacific Salmon Smolts in Rivers and Coastal Marine Waters
Source: PLoS One. 2010 Sep 23;5(9):e12916. doi: 10.1371/journal.pone.0012916 (PMC2944838; doi:10.1371/journal.pone.0012916)

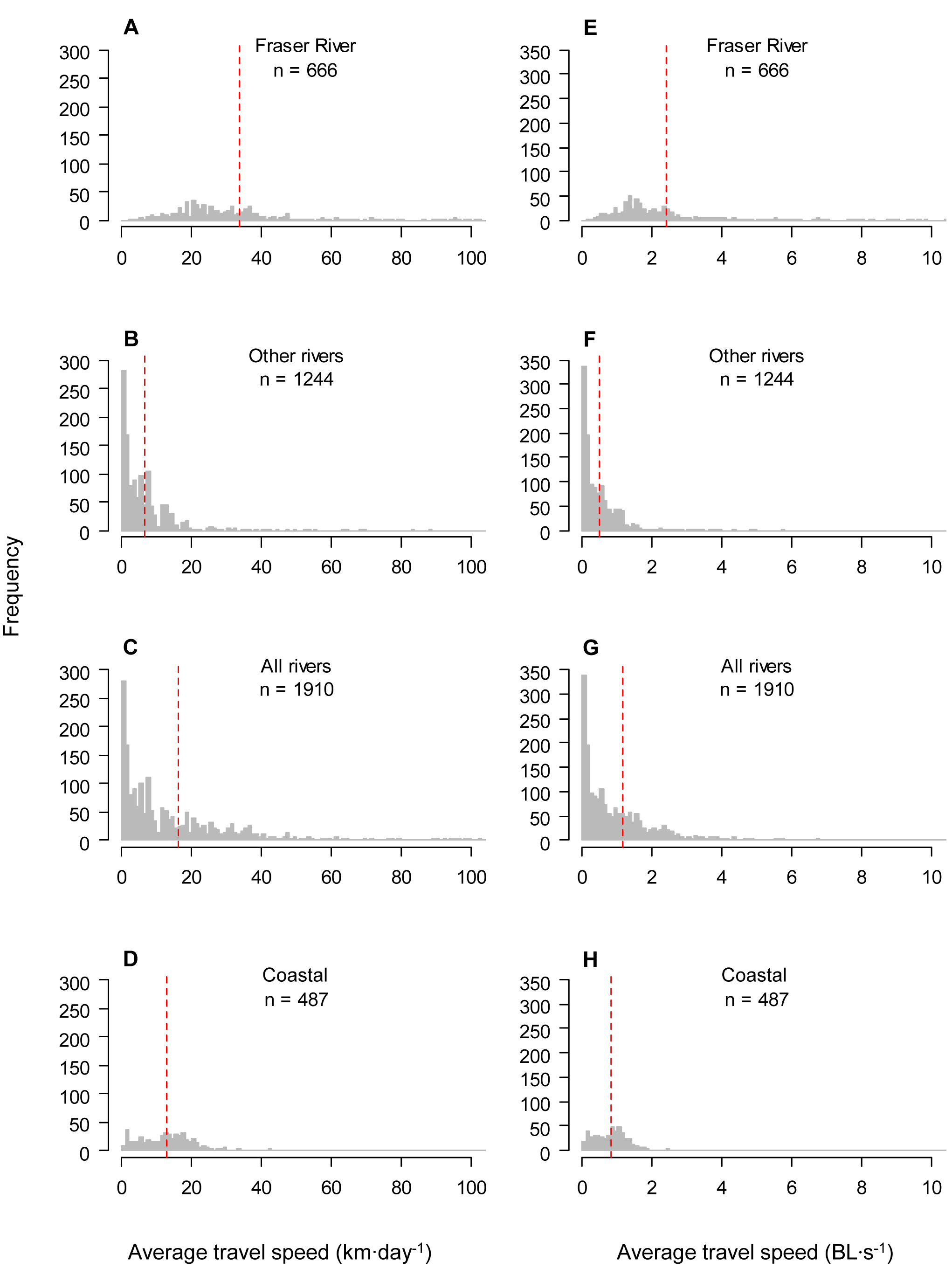

Supplement: Figure S1 — Histograms of travel speeds of tagged fish during the downstream and coastal migrations under alternate travel speed measures. Panels A–D show absolute speeds, while panels E–H show length-adjusted speeds. Frequency distributions are truncated at 100 km⋅d−1 and 10 BL⋅s−1, as few fish had speeds faster than these. Number of fish is indicated for each category. Dashed red lines show the mean travel speed in each habitat for each measure. (0.26 MB TIF) [file pone.0012916.s005.tif]

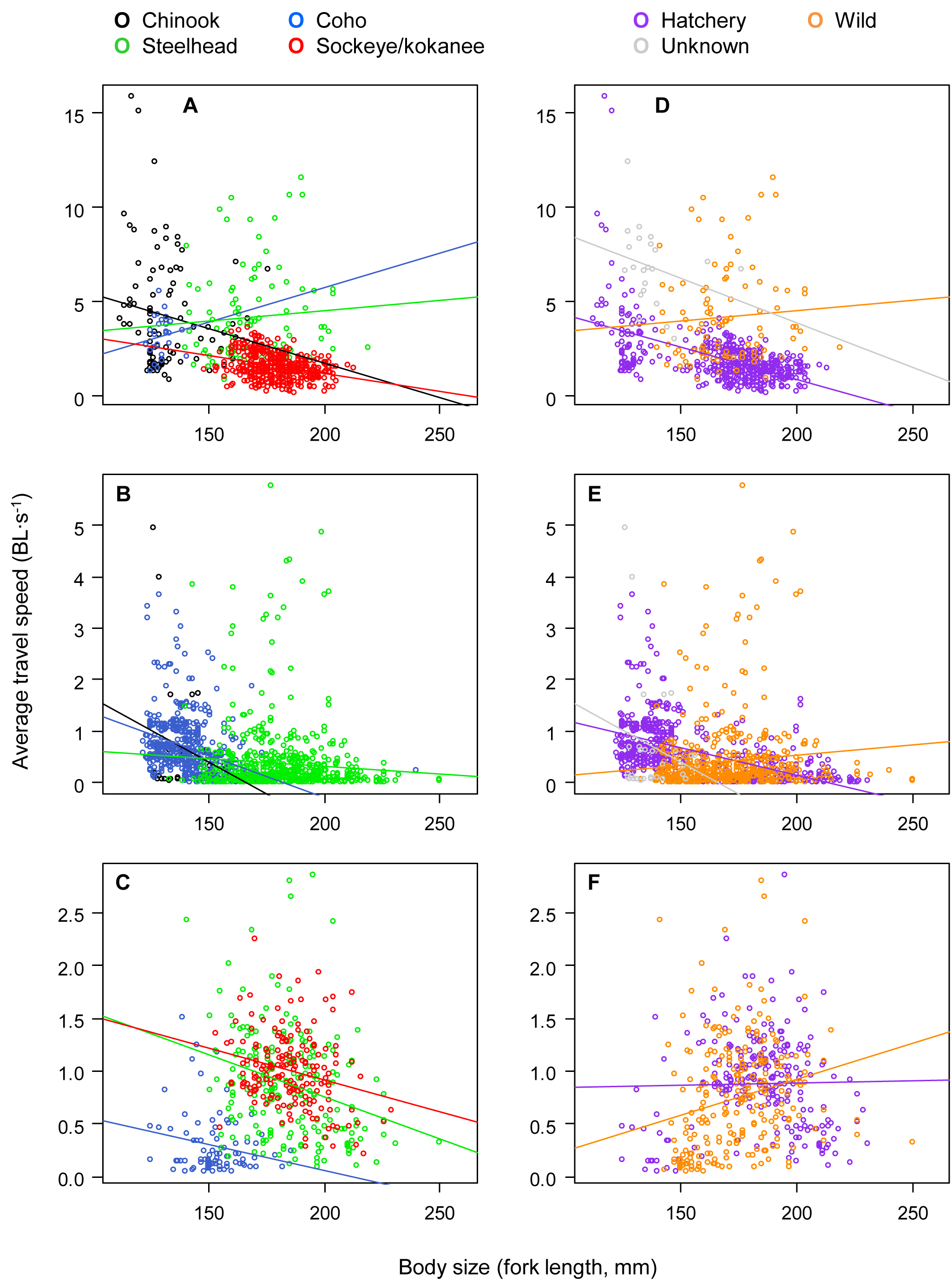

Supplement: Figure S2 — Average length-adjusted travel speeds vs. body length at time of tagging. Panels A–C show travel speed estimates separated by species, while panels D–F show estimates separated by rearing history. All estimates are in units of BL⋅s−1. Data points represent individual fish, and are separated for downstream (Fraser River: A, D; other rivers: B, E) and coastal (C, F) portions of the migration. Lines show linear regressions fit to travel speeds for each species or rearing history separately. Note the different scales on the travel time axes for Fraser River, other river, and ocean segments. (0.62 MB TIF) [file pone.0012916.s006.tif]
